# Study of charmonium production in $${b} $$-hadron decays and first evidence for the decay $${{{B}} ^0_{{s}}} \!\rightarrow \phi \phi \phi $$

**DOI:** 10.1140/epjc/s10052-017-5151-8

**Published:** 2017-09-14

**Authors:** R. Aaij, B. Adeva, M. Adinolfi, Z. Ajaltouni, S. Akar, J. Albrecht, F. Alessio, M. Alexander, S. Ali, G. Alkhazov, P. Alvarez Cartelle, A. A. Alves, S. Amato, S. Amerio, Y. Amhis, L. An, L. Anderlini, G. Andreassi, M. Andreotti, J. E. Andrews, R. B. Appleby, F. Archilli, P. d’Argent, J. Arnau Romeu, A. Artamonov, M. Artuso, E. Aslanides, G. Auriemma, M. Baalouch, I. Babuschkin, S. Bachmann, J. J. Back, A. Badalov, C. Baesso, S. Baker, V. Balagura, W. Baldini, A. Baranov, R. J. Barlow, C. Barschel, S. Barsuk, W. Barter, F. Baryshnikov, M. Baszczyk, V. Batozskaya, V. Battista, A. Bay, L. Beaucourt, J. Beddow, F. Bedeschi, I. Bediaga, A. Beiter, L. J. Bel, V. Bellee, N. Belloli, K. Belous, I. Belyaev, E. Ben-Haim, G. Bencivenni, S. Benson, S. Beranek, A. Berezhnoy, R. Bernet, A. Bertolin, C. Betancourt, F. Betti, M.-O. Bettler, M. van Beuzekom, Ia. Bezshyiko, S. Bifani, P. Billoir, A. Birnkraut, A. Bitadze, A. Bizzeti, T. Blake, F. Blanc, J. Blouw, S. Blusk, V. Bocci, T. Boettcher, A. Bondar, N. Bondar, W. Bonivento, I. Bordyuzhin, A. Borgheresi, S. Borghi, M. Borisyak, M. Borsato, F. Bossu, M. Boubdir, T. J. V. Bowcock, E. Bowen, C. Bozzi, S. Braun, T. Britton, J. Brodzicka, E. Buchanan, C. Burr, A. Bursche, J. Buytaert, S. Cadeddu, R. Calabrese, M. Calvi, M. Calvo Gomez, A. Camboni, P. Campana, D. H. Campora Perez, L. Capriotti, A. Carbone, G. Carboni, R. Cardinale, A. Cardini, P. Carniti, L. Carson, K. Carvalho Akiba, G. Casse, L. Cassina, L. Castillo Garcia, M. Cattaneo, G. Cavallero, R. Cenci, D. Chamont, M. Charles, Ph. Charpentier, G. Chatzikonstantinidis, M. Chefdeville, S. Chen, S. F. Cheung, V. Chobanova, M. Chrzaszcz, A. Chubykin, X. Cid Vidal, G. Ciezarek, P. E. L. Clarke, M. Clemencic, H. V. Cliff, J. Closier, V. Coco, J. Cogan, E. Cogneras, V. Cogoni, L. Cojocariu, P. Collins, A. Comerma-Montells, A. Contu, A. Cook, G. Coombs, S. Coquereau, G. Corti, M. Corvo, C. M. Costa Sobral, B. Couturier, G. A. Cowan, D. C. Craik, A. Crocombe, M. Cruz Torres, R. Currie, C. D’Ambrosio, F. Da Cunha Marinho, E. Dall’Occo, J. Dalseno, A. Davis, K. De Bruyn, S. De Capua, M. De Cian, J. M. De Miranda, L. De Paula, M. De Serio, P. De Simone, C. T. Dean, D. Decamp, M. Deckenhoff, L. Del Buono, H.-P. Dembinski, M. Demmer, A. Dendek, D. Derkach, O. Deschamps, F. Dettori, B. Dey, A. Di Canto, P. Di Nezza, H. Dijkstra, F. Dordei, M. Dorigo, A. Dosil Suárez, A. Dovbnya, K. Dreimanis, L. Dufour, G. Dujany, K. Dungs, P. Durante, R. Dzhelyadin, M. Dziewiecki, A. Dziurda, A. Dzyuba, N. Déléage, S. Easo, M. Ebert, U. Egede, V. Egorychev, S. Eidelman, S. Eisenhardt, U. Eitschberger, R. Ekelhof, L. Eklund, S. Ely, S. Esen, H. M. Evans, T. Evans, A. Falabella, N. Farley, S. Farry, R. Fay, D. Fazzini, D. Ferguson, G. Fernandez, A. Fernandez Prieto, F. Ferrari, F. Ferreira Rodrigues, M. Ferro-Luzzi, S. Filippov, R. A. Fini, M. Fiore, M. Fiorini, M. Firlej, C. Fitzpatrick, T. Fiutowski, F. Fleuret, K. Fohl, M. Fontana, F. Fontanelli, D. C. Forshaw, R. Forty, V. Franco Lima, M. Frank, C. Frei, J. Fu, W. Funk, E. Furfaro, C. Färber, A. Gallas Torreira, D. Galli, S. Gallorini, S. Gambetta, M. Gandelman, P. Gandini, Y. Gao, L. M. Garcia Martin, J. García Pardiñas, J. Garra Tico, L. Garrido, P. J. Garsed, D. Gascon, C. Gaspar, L. Gavardi, G. Gazzoni, D. Gerick, E. Gersabeck, M. Gersabeck, T. Gershon, Ph. Ghez, S. Gianì, V. Gibson, O. G. Girard, L. Giubega, K. Gizdov, V. V. Gligorov, D. Golubkov, A. Golutvin, A. Gomes, I. V. Gorelov, C. Gotti, E. Govorkova, R. Graciani Diaz, L. A. Granado Cardoso, E. Graugés, E. Graverini, G. Graziani, A. Grecu, R. Greim, P. Griffith, L. Grillo, B. R. Gruberg Cazon, O. Grünberg, E. Gushchin, Yu. Guz, T. Gys, C. Göbel, T. Hadavizadeh, C. Hadjivasiliou, G. Haefeli, C. Haen, S. C. Haines, B. Hamilton, X. Han, S. Hansmann-Menzemer, N. Harnew, S. T. Harnew, J. Harrison, M. Hatch, J. He, T. Head, A. Heister, K. Hennessy, P. Henrard, L. Henry, E. van Herwijnen, M. Heß, A. Hicheur, D. Hill, C. Hombach, P. H. Hopchev, Z.-C. Huard, W. Hulsbergen, T. Humair, M. Hushchyn, D. Hutchcroft, M. Idzik, P. Ilten, R. Jacobsson, J. Jalocha, E. Jans, A. Jawahery, F. Jiang, M. John, D. Johnson, C. R. Jones, C. Joram, B. Jost, N. Jurik, S. Kandybei, M. Karacson, J. M. Kariuki, S. Karodia, M. Kecke, M. Kelsey, M. Kenzie, T. Ketel, E. Khairullin, B. Khanji, C. Khurewathanakul, T. Kirn, S. Klaver, K. Klimaszewski, T. Klimkovich, S. Koliiev, M. Kolpin, I. Komarov, R. Kopecna, P. Koppenburg, A. Kosmyntseva, S. Kotriakhova, M. Kozeiha, L. Kravchuk, M. Kreps, P. Krokovny, F. Kruse, W. Krzemien, W. Kucewicz, M. Kucharczyk, V. Kudryavtsev, A. K. Kuonen, K. Kurek, T. Kvaratskheliya, D. Lacarrere, G. Lafferty, A. Lai, G. Lanfranchi, C. Langenbruch, T. Latham, C. Lazzeroni, R. Le Gac, J. van Leerdam, A. Leflat, J. Lefrançois, R. Lefèvre, F. Lemaitre, E. Lemos Cid, O. Leroy, T. Lesiak, B. Leverington, T. Li, Y. Li, Z. Li, T. Likhomanenko, R. Lindner, F. Lionetto, X. Liu, D. Loh, I. Longstaff, J. H. Lopes, D. Lucchesi, M. Lucio Martinez, H. Luo, A. Lupato, E. Luppi, O. Lupton, A. Lusiani, X. Lyu, F. Machefert, F. Maciuc, O. Maev, K. Maguire, S. Malde, A. Malinin, T. Maltsev, G. Manca, G. Mancinelli, P. Manning, J. Maratas, J. F. Marchand, U. Marconi, C. Marin Benito, M. Marinangeli, P. Marino, J. Marks, G. Martellotti, M. Martin, M. Martinelli, D. Martinez Santos, F. Martinez Vidal, D. Martins Tostes, L. M. Massacrier, A. Massafferri, R. Matev, A. Mathad, Z. Mathe, C. Matteuzzi, A. Mauri, E. Maurice, B. Maurin, A. Mazurov, M. McCann, A. McNab, R. McNulty, B. Meadows, F. Meier, D. Melnychuk, M. Merk, A. Merli, E. Michielin, D. A. Milanes, M.-N. Minard, D. S. Mitzel, A. Mogini, J. Molina Rodriguez, I. A. Monroy, S. Monteil, M. Morandin, M. J. Morello, O. Morgunova, J. Moron, A. B. Morris, R. Mountain, F. Muheim, M. Mulder, M. Mussini, D. Müller, J. Müller, K. Müller, V. Müller, P. Naik, T. Nakada, R. Nandakumar, A. Nandi, I. Nasteva, M. Needham, N. Neri, S. Neubert, N. Neufeld, M. Neuner, T. D. Nguyen, C. Nguyen-Mau, S. Nieswand, R. Niet, N. Nikitin, T. Nikodem, A. Nogay, A. Novoselov, D. P. O’Hanlon, A. Oblakowska-Mucha, V. Obraztsov, S. Ogilvy, R. Oldeman, C. J. G. Onderwater, A. Ossowska, J. M. Otalora Goicochea, P. Owen, A. Oyanguren, P. R. Pais, A. Palano, M. Palutan, A. Papanestis, M. Pappagallo, L. L. Pappalardo, C. Pappenheimer, W. Parker, C. Parkes, G. Passaleva, A. Pastore, M. Patel, C. Patrignani, A. Pearce, A. Pellegrino, G. Penso, M. Pepe Altarelli, S. Perazzini, P. Perret, L. Pescatore, K. Petridis, A. Petrolini, A. Petrov, M. Petruzzo, E. Picatoste Olloqui, B. Pietrzyk, M. Pikies, D. Pinci, A. Pistone, A. Piucci, V. Placinta, S. Playfer, M. Plo Casasus, T. Poikela, F. Polci, M. Poli Lener, A. Poluektov, I. Polyakov, E. Polycarpo, G. J. Pomery, S. Ponce, A. Popov, D. Popov, B. Popovici, S. Poslavskii, C. Potterat, E. Price, J. Prisciandaro, C. Prouve, V. Pugatch, A. Puig Navarro, G. Punzi, W. Qian, R. Quagliani, B. Rachwal, J. H. Rademacker, M. Rama, M. Ramos Pernas, M. S. Rangel, I. Raniuk, F. Ratnikov, G. Raven, F. Redi, S. Reichert, A. C. dos Reis, C. Remon Alepuz, V. Renaudin, S. Ricciardi, S. Richards, M. Rihl, K. Rinnert, V. Rives Molina, P. Robbe, A. B. Rodrigues, E. Rodrigues, J. A. Rodriguez Lopez, P. Rodriguez Perez, A. Rogozhnikov, S. Roiser, A. Rollings, V. Romanovskiy, A. Romero Vidal, J. W. Ronayne, M. Rotondo, M. S. Rudolph, T. Ruf, P. Ruiz Valls, J. J. Saborido Silva, E. Sadykhov, N. Sagidova, B. Saitta, V. Salustino Guimaraes, D. Sanchez Gonzalo, C. Sanchez Mayordomo, B. Sanmartin Sedes, R. Santacesaria, C. Santamarina Rios, M. Santimaria, E. Santovetti, A. Sarti, C. Satriano, A. Satta, D. M. Saunders, D. Savrina, S. Schael, M. Schellenberg, M. Schiller, H. Schindler, M. Schlupp, M. Schmelling, T. Schmelzer, B. Schmidt, O. Schneider, A. Schopper, H. F. Schreiner, K. Schubert, M. Schubiger, M.-H. Schune, R. Schwemmer, B. Sciascia, A. Sciubba, A. Semennikov, A. Sergi, N. Serra, J. Serrano, L. Sestini, P. Seyfert, M. Shapkin, I. Shapoval, Y. Shcheglov, T. Shears, L. Shekhtman, V. Shevchenko, B. G. Siddi, R. Silva Coutinho, L. Silva de Oliveira, G. Simi, S. Simone, M. Sirendi, N. Skidmore, T. Skwarnicki, E. Smith, I. T. Smith, J. Smith, M. Smith, l. Soares Lavra, M. D. Sokoloff, F. J. P. Soler, B. Souza De Paula, B. Spaan, P. Spradlin, S. Sridharan, F. Stagni, M. Stahl, S. Stahl, P. Stefko, S. Stefkova, O. Steinkamp, S. Stemmle, O. Stenyakin, H. Stevens, S. Stoica, S. Stone, B. Storaci, S. Stracka, M. E. Stramaglia, M. Straticiuc, U. Straumann, L. Sun, W. Sutcliffe, K. Swientek, V. Syropoulos, M. Szczekowski, T. Szumlak, S. T’Jampens, A. Tayduganov, T. Tekampe, M. Teklishyn, G. Tellarini, F. Teubert, E. Thomas, J. van Tilburg, M. J. Tilley, V. Tisserand, M. Tobin, S. Tolk, L. Tomassetti, D. Tonelli, S. Topp-Joergensen, F. Toriello, R. Tourinho Jadallah Aoude, E. Tournefier, S. Tourneur, K. Trabelsi, M. Traill, M. T. Tran, M. Tresch, A. Trisovic, A. Tsaregorodtsev, P. Tsopelas, A. Tully, N. Tuning, A. Ukleja, A. Usachov, A. Ustyuzhanin, U. Uwer, C. Vacca, V. Vagnoni, A. Valassi, S. Valat, G. Valenti, R. Vazquez Gomez, P. Vazquez Regueiro, S. Vecchi, M. van Veghel, J. J. Velthuis, M. Veltri, G. Veneziano, A. Venkateswaran, T. A. Verlage, M. Vernet, M. Vesterinen, J. V. Viana Barbosa, B. Viaud, D. Vieira, M. Vieites Diaz, H. Viemann, X. Vilasis-Cardona, M. Vitti, V. Volkov, A. Vollhardt, B. Voneki, A. Vorobyev, V. Vorobyev, C. Voß, J. A. de Vries, C. Vázquez Sierra, R. Waldi, C. Wallace, R. Wallace, J. Walsh, J. Wang, D. R. Ward, H. M. Wark, N. K. Watson, D. Websdale, A. Weiden, M. Whitehead, J. Wicht, G. Wilkinson, M. Wilkinson, M. Williams, M. P. Williams, M. Williams, T. Williams, F. F. Wilson, J. Wimberley, M. A. Winn, J. Wishahi, W. Wislicki, M. Witek, G. Wormser, S. A. Wotton, K. Wraight, K. Wyllie, Y. Xie, Z. Xu, Z. Yang, Z. Yang, Y. Yao, H. Yin, J. Yu, X. Yuan, O. Yushchenko, K. A. Zarebski, M. Zavertyaev, L. Zhang, Y. Zhang, A. Zhelezov, Y. Zheng, X. Zhu, V. Zhukov, S. Zucchelli, LHCb Collaboration

**Affiliations:** 1 Centro Brasileiro de Pesquisas Físicas (CBPF), Rio de Janeiro, Brazil; 2 Universidade Federal do Rio de Janeiro (UFRJ), Rio de Janeiro, Brazil; 3 Center for High Energy Physics, Tsinghua University, Beijing, China; 4 LAPP, Université Savoie Mont-Blanc, CNRS/IN2P3, Annecy-Le-Vieux, France; 5 Clermont Université, Université Blaise Pascal, CNRS/IN2P3, LPC, Clermont-Ferrand, France; 6 CPPM, Aix-Marseille Université, CNRS/IN2P3, Marseille, France; 7 LAL, Université Paris-Sud, CNRS/IN2P3, Orsay, France; 8 LPNHE, Université Pierre et Marie Curie, Université Paris Diderot, CNRS/IN2P3, Paris, France; 9 I. Physikalisches Institut, RWTH Aachen University, Aachen, Germany; 10 Fakultät Physik, Technische Universität Dortmund, Dortmund, Germany; 11 Max-Planck-Institut für Kernphysik (MPIK), Heidelberg, Germany; 12 Physikalisches Institut, Ruprecht-Karls-Universität Heidelberg, Heidelberg, Germany; 13 School of Physics, University College Dublin, Dublin, Ireland; 14 Sezione INFN di Bari, Bari, Italy; 15 Sezione INFN di Bologna, Bologna, Italy; 16 Sezione INFN di Cagliari, Cagliari, Italy; 17 Universita e INFN, Ferrara, Ferrara, Italy; 18 Sezione INFN di Firenze, Florence, Italy; 19 Laboratori Nazionali dell’INFN di Frascati, Frascati, Italy; 20 Sezione INFN di Genova, Genoa, Italy; 21 Universita and INFN, Milano Bicocca, Milan, Italy; 22 Sezione di Milano, Milan, Italy; 23 Sezione INFN di Padova, Padua, Italy; 24 Sezione INFN di Pisa, Pisa, Italy; 25 Sezione INFN di Roma Tor Vergata, Rome, Italy; 26 Sezione INFN di Roma La Sapienza, Rome, Italy; 27 Henryk Niewodniczanski Institute of Nuclear Physics, Polish Academy of Sciences, Kraków, Poland; 28 Faculty of Physics and Applied Computer Science, AGH-University of Science and Technology, Kraków, Poland; 29 National Center for Nuclear Research (NCBJ), Warsaw, Poland; 30 Horia Hulubei National Institute of Physics and Nuclear Engineering, Bucharest-Magurele, Romania; 31 Petersburg Nuclear Physics Institute (PNPI), Gatchina, Russia; 32 Institute of Theoretical and Experimental Physics (ITEP), Moscow, Russia; 33 Institute of Nuclear Physics, Moscow State University (SINP MSU), Moscow, Russia; 34 Institute for Nuclear Research of the Russian Academy of Sciences (INR RAN), Moscow, Russia; 35 Yandex School of Data Analysis, Moscow, Russia; 36 Budker Institute of Nuclear Physics (SB RAS), Novosibirsk, Russia; 37 Institute for High Energy Physics (IHEP), Protvino, Russia; 38 ICCUB, Universitat de Barcelona, Barcelona, Spain; 39 Universidad de Santiago de Compostela, Santiago de Compostela, Spain; 40 European Organization for Nuclear Research (CERN), Geneva, Switzerland; 41 Institute of Physics, Ecole Polytechnique Fédérale de Lausanne (EPFL), Lausanne, Switzerland; 42 Physik-Institut, Universität Zürich, Zurich, Switzerland; 43 Nikhef National Institute for Subatomic Physics, Amsterdam, The Netherlands; 44 Nikhef National Institute for Subatomic Physics and VU University Amsterdam, Amsterdam, The Netherlands; 45 NSC Kharkiv Institute of Physics and Technology (NSC KIPT), Kharkiv, Ukraine; 46 Institute for Nuclear Research of the National Academy of Sciences (KINR), Kiev, Ukraine; 47 University of Birmingham, Birmingham, UK; 48 H.H. Wills Physics Laboratory, University of Bristol, Bristol, UK; 49 Cavendish Laboratory, University of Cambridge, Cambridge, UK; 50 Department of Physics, University of Warwick, Coventry, UK; 51 STFC Rutherford Appleton Laboratory, Didcot, UK; 52 School of Physics and Astronomy, University of Edinburgh, Edinburgh, UK; 53 School of Physics and Astronomy, University of Glasgow, Glasgow, UK; 54 Oliver Lodge Laboratory, University of Liverpool, Liverpool, UK; 55 Imperial College London, London, UK; 56 School of Physics and Astronomy, University of Manchester, Manchester, UK; 57 Department of Physics, University of Oxford, Oxford, UK; 58 Massachusetts Institute of Technology, Cambridge, MA USA; 59 University of Cincinnati, Cincinnati, OH USA; 60 University of Maryland, College Park, MD USA; 61 Syracuse University, Syracuse, NY USA; 62 Pontifícia Universidade Católica do Rio de Janeiro (PUC-Rio), Rio de Janeiro, Brazil; 63 University of Chinese Academy of Sciences, Beijing, China; 64 School of Physics and Technology, Wuhan University, Wuhan, China; 65 Institute of Particle Physics, Central China Normal University, Wuhan, Hubei China; 66 Departamento de Fisica, Universidad Nacional de Colombia, Bogota, Colombia; 67 Institut für Physik, Universität Rostock, Rostock, Germany; 68 National Research Centre Kurchatov Institute, Moscow, Russia; 69 Instituto de Fisica Corpuscular, Centro Mixto Universidad de Valencia-CSIC, Valencia, Spain; 70 Van Swinderen Institute, University of Groningen, Groningen, The Netherlands; 71 CERN, 1211 Geneva 23, Switzerland

## Abstract

Using decays to $$\phi $$-meson pairs, the inclusive production of charmonium states in $${b} $$-hadron decays is studied with *pp* collision data corresponding to an integrated luminosity of $$3.0 {\,\mathrm{fb}}^{-1} $$, collected by the LHCb experiment at centre-of-mass energies of 7 and 8 TeV. Denoting by $${\mathcal {B}} _C \equiv {\mathcal {B}} ( {{b}} \!\rightarrow C X ) \times {\mathcal {B}} ( C\!\rightarrow \phi \phi )$$ the inclusive branching fraction of a $${{b}} $$ hadron to a charmonium state *C* that decays into a pair of $$\phi $$ mesons, ratios $$R^{C_1}_{C_2}\equiv {\mathcal {B}} _{C_1} / {\mathcal {B}} _{C_2}$$ are determined as $$R^{{\upchi _{{{c}} 0}}}_{{\eta _{{c}}} (1S)} = 0.147 \pm 0.023 \pm 0.011$$, $$R^{{\upchi _{{{c}} 1}}}_{{\eta _{{c}}} (1S)} = 0.073 \pm 0.016 \pm 0.006$$, $$R^{{\upchi _{{{c}} 2}}}_{{\eta _{{c}}} (1S)} = 0.081 \pm 0.013 \pm 0.005$$, $$R^{{\upchi _{{{c}} 1}}}_{{\upchi _{{{c}} 0}}} = 0.50 \pm 0.11 \pm 0.01$$, $$R^{{\upchi _{{{c}} 2}}}_{{\upchi _{{{c}} 0}}} = 0.56 \pm 0.10 \pm 0.01$$ and $$R^{{\eta _{{c}}} (2S)}_{{\eta _{{c}}} (1S)} = 0.040 \pm 0.011 \pm 0.004$$. Here and below the first uncertainties are statistical and the second systematic. Upper limits at 90% confidence level for the inclusive production of *X*(3872), *X*(3915) and $${\upchi _{{{c}} 2}} (2P)$$ states are obtained as $$R^{X(3872)}_{{\upchi _{{{c}} 1}}} < 0.34$$, $$R^{X(3915)}_{{\upchi _{{{c}} 0}}} < 0.12$$ and $$R^{{\upchi _{{{c}} 2}} (2P)}_{{\upchi _{{{c}} 2}}} < 0.16$$. Differential cross-sections as a function of transverse momentum are measured for the $${\eta _{{c}}} (1S)$$ and $$\chi _c$$ states. The branching fraction of the decay $${{{B}} ^0_{{s}}} \!\rightarrow \phi \phi \phi $$ is measured for the first time, $${\mathcal {B}} ( {{{B}} ^0_{{s}}} \!\rightarrow \phi \phi \phi ) = (2.15 \pm 0.54 \pm 0.28 \pm 0.21_{{\mathcal {B}}}) \times 10^{-6}$$. Here the third uncertainty is due to the branching fraction of the decay $${{{B}} ^0_{{s}}} \!\rightarrow \phi \phi $$, which is used for normalization. No evidence for intermediate resonances is seen. A preferentially transverse $$\phi $$ polarization is observed. The measurements allow the determination of the ratio of the branching fractions for the $${\eta _{{c}}} (1S)$$ decays to $$\phi \phi $$ and $${{p}} {\overline{{{p}}}} $$ as $${\mathcal {B}} ( {\eta _{{c}}} (1S)\!\rightarrow \phi \phi )/{\mathcal {B}} ( {\eta _{{c}}} (1S)\!\rightarrow {{p}} {\overline{{{p}}}} ) = 1.79 \pm 0.14\pm 0.32$$.

## Introduction

The production of the $$J^{PC} = 1^{--}$$ charmonium states has been extensively studied using decays to clean dilepton final states. Other states such as those from the $$\chi _c$$ family can be accessed via the radiative transition to $${{J}/\uppsi }$$. Studies of the production of the non-$$1^{--}$$ charmonium states can be performed by reconstructing their decays to fully hadronic final states [1]. This paper reports a measurement of the inclusive production rates of the $$\eta _{{c}} $$ and $$\chi _c$$ states in $${b} $$-hadron decays, $${{b}} \!\rightarrow {\eta _{{c}}} X $$ and $${{b}} \!\rightarrow \chi _c X $$, using charmonia decays to a pair of $$\phi $$ mesons. In addition, the first evidence for the decay $${{{B}} ^0_{{s}}} \!\rightarrow \phi \phi \phi $$ is reported.

Results on inclusive charmonium production in $${b} $$-hadron decays are available from $${e} ^+{e} ^-$$ experiments operating at centre-of-mass energies around the $${\Upsilon {(4S)}}$$ and $${\Upsilon {(5S)}}$$ resonances, studying mixtures of $${{{B}} ^+} $$ and $${{B}} ^0$$ mesons^1^ (light mixture) or $${{{B}} ^+} $$, $${{B}} ^0$$ and $${{B}} ^0_{{s}} $$ mesons, respectively. Mixtures of all $${b} $$-hadrons ($${{{B}} ^+} $$, $${{B}} ^0$$, $${{B}} ^0_{{s}} $$, $${{B}} _{{c}} ^+$$ and $${b} $$-baryons) have been studied at LEP, the Tevatron and the LHC. The world average values for charmonium branching fractions from the light mixture are dominated by results from the CLEO  [2, 3], Belle  [4] and BaBar  [5] collaborations. For the $${{J}/\uppsi }$$, $$\uppsi {(2S)}$$ and $$\upchi _{{{c}} 1}$$ states the measured branching fractions are consistent within uncertainties. The new Belle result for the $${{b}} \!\rightarrow {\upchi _{{{c}} 2}} X $$ branching fraction [4], which supersedes the previous measurement [6], is below the BaBar result [5] by more than 2.5 standard deviations, while the CLEO collaboration does not observe a statistically significant $${{b}} \!\rightarrow {\upchi _{{{c}} 2}} X $$ signal [3]. An upper limit on the inclusive production rate of $${\eta _{{c}}} (1S)$$ mesons in the light mixture, $${\mathcal {B}} ( {{B}} \rightarrow {\eta _{{c}}} (1S) X ) < 9 \times 10^{-3}$$ at $$90 \%$$ confidence level (CL), was reported by CLEO  [7].

The branching fractions of $${b} $$-hadron decays to final states including a $${{J}/\uppsi }$$ or $$\uppsi {(2S)}$$ charmonium state, where all $${b} $$-hadron species are involved, are known with uncertainties of around 10%, with the world averages dominated by the measurements performed at LEP  [8–10]. The ratio of $${{b}} \!\rightarrow {\uppsi {(2S)}} X $$ and $${{b}} \!\rightarrow {{{J}/\uppsi }} X $$ yields has been measured at the LHC by the LHCb, CMS and ATLAS collaborations with a precision of around 5% [11–13]. The only available results for the $$\upchi _{c}$$ family are the $$\upchi _{{{c}} 1}$$ inclusive production rates in $${b} $$-hadron decays measured by the DELPHI and L3 collaborations [8, 9], with an average value of $${\mathcal {B}} ( {{b}} \rightarrow {\upchi _{{{c}} 1}} X) = (14 \pm 4) \times 10^{-3}$$ [14]. Recently, LHCb measured the $${\eta _{{c}}} (1S)$$ production rate, $${\mathcal {B}} ( {{b}} \rightarrow {\eta _{{c}}} (1S) X) = (4.88 \pm 0.64 \pm 0.29 \pm 0.67_{{\mathcal {B}}}) \times 10^{-3}$$, where the third uncertainty is due to uncertainties on the $${{J}/\uppsi }$$ inclusive branching fraction from $${b} $$-hadron decays and the branching fractions of the decays of $${{J}/\uppsi }$$ and $${\eta _{{c}}} (1S)$$ to the $${p} $$ $$\overline{{{p}}}$$ final state [15].

While experimentally the reconstruction of charmonia from $${b} $$-hadron decays allows an efficient control of combinatorial background with respect to charmonium candidates produced in the $${p} $$ $${p} $$ collision vertex via hadroproduction or in the decays of heavier resonances (prompt charmonium), inclusive $${b} $$-hadron decays to charmonia are theoretically less clean. Since a description of the strong interaction dynamics in $${b} $$-hadron inclusive decays improves with respect to exclusive decays due to consideration of more final states, and the formation of charmonium proceeds through a short-distance process, a factorization of a $$c\bar{c}$$ pair production and its hadronization in a given charmonium state becomes a reasonable assumption [16]. The relative inclusive production of $$\upchi _{c}$$ states in $${b} $$-hadron decays provides a clean test of charmonia production models. For example, the colour evaporation model predicts a $${\upchi _{{{c}} 2}}/ {\upchi _{{{c}} 1}} $$ production ratio of 5 / 3 [17], while the perturbative QCD-based computation predicts that the V-A current, which is responsible for the $${b} $$ decays, forbids the $$\upchi _{{{c}} 2}$$ and $$\upchi _{{{c}} 0}$$ production at leading order. In the non-relativistic QCD (NRQCD) framework [18–20], the colour-octet contributions have to be included, predicting the rates to be proportional to $$(2J + 1)$$ for the $${\upchi _{c}} _J$$ states. The NRQCD framework can be applied to both prompt charmonium production and secondary production from $${b} $$-hadron decays and the comparison between these two production mechanisms can provide a valuable test of this theoretical framework.

In this paper we report the first measurements of inclusive $$\chi _c$$ and $${\eta _{{c}}} (2S)$$ production rates in $${b} $$-hadron decays using charmonium decays to hadronic final states in the high-multiplicity environment of a hadron collider. Experimentally, charmonium candidates from $${b} $$-hadron decays are distinguished from prompt charmonia by exploiting the $${b} $$-hadron decay time and reconstructing a $${b} $$-hadron (and charmonium) decay vertex well separated from the primary vertex where the $${b} $$-hadron candidate was produced. The charmonium states are reconstructed via their decays to a $$\phi \phi $$ final state. The $${\eta _{{c}}} (1S)$$ production followed by the decay $${\eta _{{c}}} (1S) \rightarrow \phi \phi $$ is used for normalization, so that systematic uncertainties partially cancel in the ratios. As a by-product of the production rate measurements, the masses of the $${\eta _{{c}}} (1S)$$, $$\upchi _{{{c}} 0}$$, $$\upchi _{{{c}} 1}$$, $$\upchi _{{{c}} 2}$$ and $${\eta _{{c}}} (2S)$$ charmonium states and the natural width of the $${\eta _{{c}}} (1S)$$ meson are determined.

The $${{B}} ^0_{{s}} $$ decay to the $$\phi \phi $$ final state has been observed by the CDF collaboration [21] and recently precisely measured by the LHCb collaboration [22], where it was also used to search for $$C\!P$$-violating asymmetries [23]. In the Standard Model (SM) the amplitude for the decay $${{{B}} ^0_{{s}}} \rightarrow \phi \phi $$ is dominated by a loop diagram. Experimental verification of the partial width, polarization amplitudes and triple-product asymmetries of the $${{{B}} ^0_{{s}}} \rightarrow \phi \phi $$ decay probes the QCD contribution to the weak processes described by nonfactorizable penguin diagrams [24, 25], and contributions from particles beyond the SM to the penguin loops [26–30]. A three-body $${{{B}} ^0_{{s}}} \rightarrow \phi \phi \phi $$ decay leads to a final state with six strange quarks. In the SM it is described by the penguin diagram of the $${{{B}} ^0_{{s}}} \rightarrow \phi \phi $$ decay with the creation of an additional $${{s}} {\overline{{{s}}}} $$ quark pair. The $${{{B}} ^0_{{s}}} \rightarrow \phi \phi \phi $$ decay can also receive contributions from an intermediate charmonium state decaying to a $$\phi \phi $$ state. Here we report first evidence for the $${{{B}} ^0_{{s}}} \rightarrow \phi \phi \phi $$ decay and study its resonance structure. The branching fraction of this decay is determined relative to the branching fraction $${\mathcal {B}} ( {{{B}} ^0_{{s}}} \rightarrow \phi \phi )$$ [22]. To cross-check the technique exploited in this paper, the value of $${\mathcal {B}} ( {{{B}} ^0_{{s}}} \rightarrow \phi \phi )$$ is also determined relative to the $${\eta _{{c}}} (1S)$$ production rate. Finally, the ratio of the branching fractions for the decays $${\eta _{{c}}} (1S) \rightarrow \phi \phi $$ and $${\eta _{{c}}} (1S) \rightarrow {{p}} {\overline{{{p}}}} $$ is determined, using additional external information.

The LHCb detector and data sample used for the analysis are presented in Sect. 2. Section 3 explains the selection details and the signal extraction technique. Inclusive production of charmonium states in $${b} $$-hadron decays is discussed in Sect. 4. In Sect. 5 measurements of the $${\eta _{{c}}} (1S)$$ mass and natural width are described. First evidence for the $${{{B}} ^0_{{s}}} \rightarrow \phi \phi \phi $$ decay is reported in Sect. 6. The main results of the paper are summarized in Sect. 7.

## LHCb detector and data sample

The LHCb detector [31, 32] is a single-arm forward spectrometer covering the pseudorapidity range $$2<\eta <5$$, designed for the study of particles containing $${b} $$ or $${c} $$ quarks. The detector includes a high-precision tracking system consisting of a silicon-strip vertex detector surrounding the *pp* interaction region, a large-area silicon-strip detector located upstream of a dipole magnet with a bending power of about $$4{\mathrm{\,Tm}}$$, and three stations of silicon-strip detectors and straw drift tubes placed downstream of the magnet. The tracking system provides a measurement of momentum, $$p$$, of charged particles with a relative uncertainty that varies from 0.5% at low momentum^2^ to 1.0% at 200$${\,\mathrm{GeV}}$$. The minimum distance of a track to a primary vertex (PV), the impact parameter (IP), is measured with a resolution of $$(15+29/p_{\mathrm{T}}){\,\upmu \mathrm{m}} $$, where $$p_{\mathrm{T}}$$ is the component of the momentum transverse to the beam, in $${\,\mathrm{GeV}}$$. Different types of charged hadrons are distinguished using information from two ring-imaging Cherenkov detectors. Photons, electrons and hadrons are identified by a calorimeter system consisting of scintillating-pad and preshower detectors, an electromagnetic calorimeter and a hadronic calorimeter. Muons are identified by a system composed of alternating layers of iron and multiwire proportional chambers. The online event selection is performed by a trigger, which consists of a hardware stage, based on information from the calorimeter and muon systems, followed by a software stage, which applies a full event reconstruction.

The analysis is based on $${p} $$ $${p} $$ collision data recorded by the LHCb experiment at a centre-of-mass energy $$\sqrt{s} = 7 {~\mathrm{TeV}}$$, corresponding to an integrated luminosity of $$1.0 {\,\mathrm{fb}}^{-1} $$, and at $$\sqrt{s} = 8 {~\mathrm{TeV}}$$, corresponding to an integrated luminosity of $$2.0 {\,\mathrm{fb}}^{-1} $$. Events enriched in signal decays are selected by the hardware trigger, based on the presence of a single deposit of high transverse energy in the calorimeter. The subsequent software trigger selects events with displaced vertices formed by charged particles having a good track-fit quality, transverse momentum larger than 0.5$${\,\mathrm{GeV}}$$, and that are incompatible with originating from any PV [23]. Charged kaon candidates are identified using the information from the Cherenkov and tracking detectors. Two oppositely charged kaon candidates having an invariant mass within $$\pm 11 {\,\mathrm{MeV}} $$ of the known mass of the $$\phi $$ meson are required to form a good quality vertex.

Precise mass measurements require a momentum-scale calibration. The procedure [33] uses $${{{J}/\uppsi }} \rightarrow {\upmu ^+} {\upmu ^-} $$ decays to cross-calibrate a relative momentum scale between different data-taking periods. The absolute scale is determined using $${{{{B}} ^+}} \rightarrow {{{J}/\uppsi }} {{\mathrm{K}} ^+} $$ decays with known particle masses as input [14]. The final calibration is checked with a variety of fully reconstructed quarkonium, $${{{B}} ^+} $$ and $${\mathrm{K}} ^0_{\mathrm{S}}$$ decays. No residual bias is observed within the experimental resolution.

In the simulation, *pp* collisions are generated using Pythia  [34, 35] with a specific LHCb configuration [36]. Decays of hadronic particles are described by EvtGen  [37], in which final-state radiation is generated using Photos  [38]. The interaction of the generated particles with the detector material and its response are implemented using the Geant4 toolkit [39, 40] as described in Ref. [41]. Simulated samples of $${\eta _{{c}}} $$ and $$\chi _c$$ mesons decaying to the $$\phi \phi $$ final state, and $${{B}} ^0_{{s}} $$ decaying to two and three $$\phi $$ mesons, are used to estimate efficiency ratios and to evaluate systematic uncertainties. Charmonium and $${{{B}} ^0_{{s}}} \rightarrow \phi \phi \phi $$ decays are generated with uniform phase-space density, while $${{{B}} ^0_{{s}}} \rightarrow \phi \phi $$ decays are generated according to the amplitudes from Ref. [21].

## Selection criteria and signal extraction

The signal selection is largely performed at the trigger level. The offline analysis selects combinations of two or three $$\phi $$ candidates that are required to form a good-quality common vertex displaced from the primary vertex. A good separation between the two vertices ($$\chi ^2 > 100$$) is required, reducing the contribution from charmonia produced directly at the primary vertex to below $$1 \%$$. Pairs of $$\phi $$ mesons originating from different $${b} $$-hadrons produced in the same beam crossing event are suppressed by the requirement of a good-quality common vertex. Detector acceptance and selection requirements, and in particular the requirement of the kaon $$p_{\mathrm{T}}$$ to exceed 0.5$${\,\mathrm{GeV}}$$, significantly suppress $$\phi \phi $$ combinations with $$p_{\mathrm{T}}$$ below $$4{\,\mathrm{GeV}} $$.

**Fig. 1:**
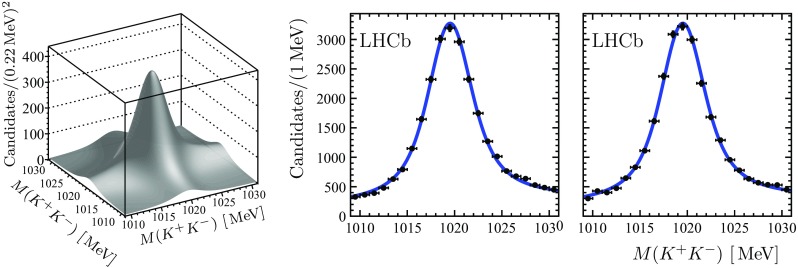
Result of the 2D fit to the $2 ( K^+ K^- )$ invariant mass distribution along with the projections to the ${{\mathrm{K}} ^+} {{\mathrm{K}} ^-}$ invariant mass axes in the ${\eta _{{c}}} (1S)$ signal region

**Fig. 2:**
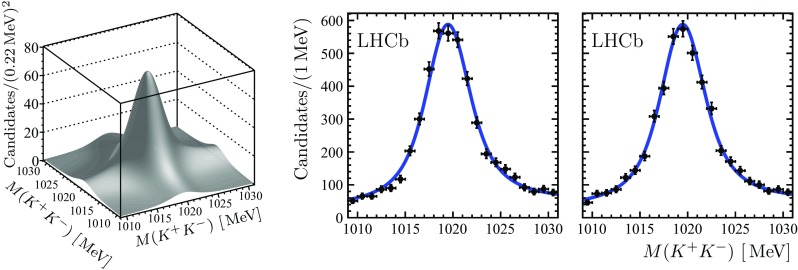
Result of the 2D fit to the $$2 ( K^+ K^- )$$ invariant mass distribution along with the projections to the $${{\mathrm{K}} ^+} {{\mathrm{K}} ^-} $$ invariant mass axes in the $${{B}} ^0_{{s}} $$ signal region

**Fig. 3:**
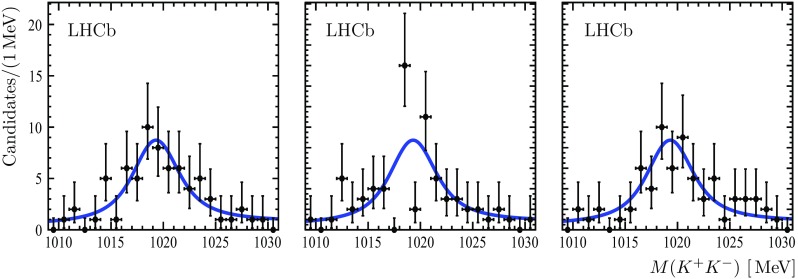
Projections to the $${{\mathrm{K}} ^+} {{\mathrm{K}} ^-} $$ invariant mass axes of the 3D fit to the $$3 ( K^+ K^- )$$ invariant mass distribution in the $${{B}} ^0_{{s}} $$ signal region

Two-dimensional (2D) or three-dimensional (3D) extended unbinned maximum likelihood fits, corresponding to the two or three randomly ordered $$K^+ K^-$$ combinations, are performed in bins of the invariant mass of the four-kaon and six-kaon combinations, denoted as $$2 ( K^+ K^- )$$ or $$3 ( K^+ K^- )$$, respectively, to determine the numbers of $$\phi \phi $$ and $$\phi \phi \phi $$ combinations. The 2D fit accounts for the signal, $$\phi \phi $$, and background, $$\phi \, ( K^+ K^- )$$ and $$2 ( K^+ K^- )$$, components, while the 3D fit accounts for the signal, $$\phi \phi \phi $$, and background, $$\phi \phi \, ( K^+ K^- )$$, $$\phi \, 2( K^+ K^- )$$ and $$3 ( K^+ K^- )$$, components. A $$\phi $$ signal is described by the convolution of a Breit–Wigner function and a sum of two Gaussian functions with a common mean. The ratio of the two Gaussian widths and the fraction of the narrower Gaussian are taken from simulation. The contribution from combinatorial background, due to $${{\mathrm{K}} ^+} {{\mathrm{K}} ^-} $$ pairs not originating from the decay of a $$\phi $$ meson, is assumed to be flat. In addition, a threshold function to account for the available phase-space in the $${{\mathrm{K}} ^+} {{\mathrm{K}} ^-} $$ system is introduced for both signal and combinatorial background. While no visible contribution from the $$f_0 (980)$$ resonance decaying into a $$K^+ K^-$$ pair is observed in the $$2 ( K^+ K^- )$$ or $$3 ( K^+ K^- )$$ combinations, a potential effect due to contributions from such decays is considered as a source of systematic uncertainty. Figures 1 and 2 show the results of the 2D fits to the $$2 ( K^+ K^- )$$ invariant mass distributions along with the projections to the $${{\mathrm{K}} ^+} {{\mathrm{K}} ^-} $$ invariant mass axes in the $${\eta _{{c}}} (1S)$$ and $${{B}} ^0_{{s}} $$ signal regions, $$2.91 - 3.06 {\,\mathrm{GeV}} $$ and $$5.30 - 5.43 {\,\mathrm{GeV}} $$. Figure 3 shows the projections to the $${{\mathrm{K}} ^+} {{\mathrm{K}} ^-} $$ invariant mass axes of the 3D fit to the $$3 ( K^+ K^- )$$ invariant mass distribution in the $${{B}} ^0_{{s}} $$ signal region. While using the known value for the natural width of the $$\phi $$ resonance [14], the $$\phi $$ mass and the remaining resolution parameter are determined from the fits in the enlarged signal $$\phi \phi $$ and $$\phi \phi \phi $$ invariant mass regions. In the 2D and 3D fits in the bins of $$\phi \phi $$ and $$\phi \phi \phi $$ invariant mass the $$\phi $$ mass and the resolution parameter are then fixed to the values determined in the enlarged signal regions.

Unless they are extracted from the 2D or 3D fits, throughout the paper the error bars shown in the histograms are determined as follows: the upper (lower) error bar assigned to a given bin content *N* is defined by the expectation value $\lambda$ of the Poissonian distribution giving 16% probability to observe the number of events *N* or less (more). When obtained from the 2D or 3D fits the histogram bin contents are constrained to be positive, with error bars defined by the range in the allowed region where the best fit negative-log likelihood value is within half a unit from the global minimum.

In the following, production ratios are determined from the signal yields obtained from the fits of the $\phi \phi$ or $\phi \phi \phi$ invariant mass spectra. The relative efficiencies are taken into account to determine the ratio of the branching fractions of the corresponding decays. Signal yields corresponding to the data samples accumulated at $\sqrt{s} = 7$ and $8 {~\mathrm{TeV}}$ are found to be compatible. Unless otherwise specified, the results below are based on the analysis of the combined data sample.

## Charmonium production in decays to $\varvec{\phi \phi }$

### Charmonium yields

**Fig. 4:**
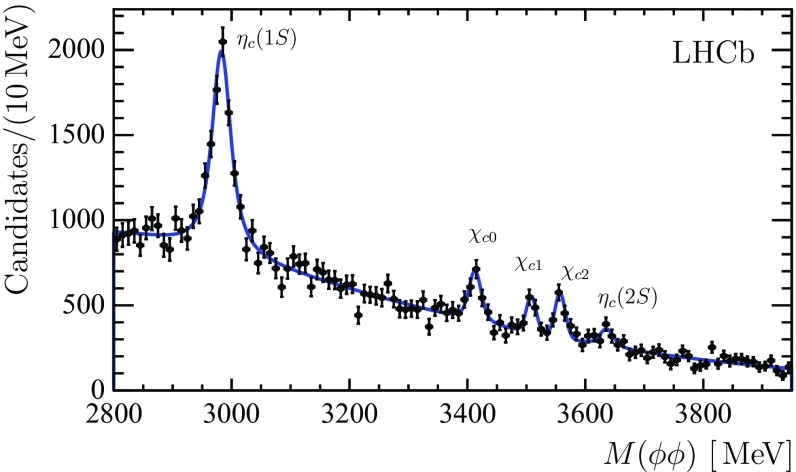
Distribution of the invariant mass of $\phi \phi$ combinations. The number of candidates in each bin is obtained from the corresponding 2D fit. The peaks corresponding to the ${{c}} {\overline{{{c}}}}$ resonances are marked on the plot. The signal yields are given in Table 1

Figure 4 shows the invariant mass spectrum of the $$\phi \phi $$ combinations, where the content of each bin is a result of a 2D fit to the two $${{\mathrm{K}} ^+} {{\mathrm{K}} ^-} $$ invariant-mass combinations. A binned $$\chi ^2$$ fit to the spectrum is used to determine the contributions from the $${\eta _{{c}}} (1S)$$ and $${\eta _{{c}}} (2S)$$ mesons, and the $$\upchi _{{{c}} 0}$$, $$\upchi _{{{c}} 1}$$ and $$\upchi _{{{c}} 2}$$ mesons. The charmonium-like states *X*(3872), *X*(3915) and $${\upchi _{{{c}} 2}} (2P)$$ with masses and natural widths from Ref. [14] are taken into account in alternative fits in order to evaluate systematic uncertainties, as well as to obtain upper limits on the inclusive production of these states in $${b} $$-hadron decays. Each resonance is described by the convolution of a relativistic Breit–Wigner function and a sum of two Gaussian functions with a common mean. The combinatorial background, *i.e.* contributions due to random combinations of two true $$\phi $$ mesons, is described by the product of a first-order polynomial with an exponential function and a threshold factor. The natural width of the $${\eta _{{c}}} (1S)$$ state is a free parameter in the fit, while the natural widths of the $${\eta _{{c}}} (2S)$$ and the $$\chi _c$$ states, which have lower signal yields, are fixed to their known values [14]. Possible variations of the $${\eta _{{c}}} (2S)$$ production rate depending on its natural width, $$\Gamma _{{\eta _{{c}}} (2S)}$$, are explored by providing the result as a function of the assumed natural width. The ratio of the two Gaussian widths and the fraction of the narrow Gaussian are fixed from the simulation. The mass resolution for different charmonium resonances is scaled according to the energy release, so that a single free parameter in the $$\phi \phi $$ invariant mass fit accounts for the detector resolution. This scaling of the mass resolution for different charmonium states has been validated using simulation.

**Table 1:** Signal yields with statistical uncertainties of the fit to the spectrum of the $\phi \phi$ invariant mass

Resonance	Signal yield
${\eta _{{c}}} (1S)$	$6476\pm 418$
$\upchi _{{{c}} 0}$	$933\pm 128$
$\upchi _{{{c}} 1}$	$460\pm 89$
$\upchi _{{{c}} 2}$	$611\pm 97$
${\eta _{{c}}} (2S)$	$365\pm 100$

**Table 2:** The ratio of charmonium signal yields with respect to the ${\eta _{{c}}} (1S)$ yield and between pairs of $\upchi _{c}$ states. The first uncertainties are statistical and the second systematic

Resonances	Signal yield ratio
$N_{{\upchi _{{{c}} 0}}} / N_{{\eta _{{c}}} (1S)}$	$0.144 \pm 0.022 \pm 0.011$
$N_{{\upchi _{{{c}} 1}}} / N_{{\eta _{{c}}} (1S)}$	$0.071 \pm 0.015 \pm 0.006$
$N_{{\upchi _{{{c}} 2}}} / N_{{\eta _{{c}}} (1S)}$	$0.094 \pm 0.016 \pm 0.006$
$N_{{\upchi _{{{c}} 1}}} / N_{{\upchi _{{{c}} 0}}}$	$0.494 \pm 0.107 \pm 0.012$
$N_{{\upchi _{{{c}} 2}}} / N_{{\upchi _{{{c}} 0}}}$	$0.656 \pm 0.121 \pm 0.015$
$N_{{\eta _{{c}}} (2S)} / N_{{\eta _{{c}}} (1S)}$	$0.056 \pm 0.016 \pm 0.005$

The signal yields are given in Table 1. The ratios of the resonance yields from the fit are given in Table 2, both for the ratios with respect to the ${\eta _{{c}}} (1S)$ yield and between pairs of $\upchi _{c}$ states; the systematic uncertainties are discussed below. The statistical significance for the $N_{{\eta _{{c}}} (2S)}$ signal is estimated from the $\chi ^2$-profile to be 3.7 standard deviations.

Systematic uncertainties in the ratios of the charmonium yields are estimated by considering potential contributions from other states, from imperfect modelling of detector resolution, signal resonances and background, and from the 2D fit technique. In order to evaluate the systematic uncertainty related to potential contributions from other states, signal shapes for the *X*(3872), *X*(3915), and ${\upchi _{{{c}} 2}} (2P)$ states are included in the fit. Systematic uncertainties related to detector resolution are estimated by fixing the ${\eta _{{c}}} (1S)$ mass resolution to the value determined from the simulation. In addition, systematic uncertainties associated to the impact of the detector resolution on the signal shapes are estimated by comparing the nominal fit results to those obtained using a single instead of a double Gaussian function. An uncertainty associated with the description of the mass resolution of the $\phi$ meson is estimated by fixing the resolution in the 2D fits to the value determined from simulation. The uncertainty associated with the description using the relativistic Breit–Wigner line shape [42] is estimated by varying the radial parameter of the Blatt–Weisskopf barrier factor [43] between 0.5 and $3 \, {\,\mathrm{GeV}} ^{-1}$. In order to estimate the uncertainty related to the natural width of the ${\eta _{{c}}} (2S)$ meson, the value of $\Gamma _{{\eta _{{c}}} (2S)}$ is varied within the uncertainties of the world average [14]. The uncertainty in the description of the $\chi _c$ signal peaks is estimated by fixing the $\chi _c$ masses to their known values. A reduced fit range, covering only the $\chi _c$ and ${\eta _{{c}}} (2S)$ region ($3.15{-}3.95 {\,\mathrm{GeV}}$), is used to estimate a systematic uncertainty associated to the choice of the fit range. An alternative background parametrization using a parabolic instead of a linear function is used to estimate the systematic uncertainty due to the choice of the background parametrization. A systematic uncertainty associated to the background parametrization in the 2D fits is estimated by adding slope parameters to the description of the non-$\phi$ ${{\mathrm{K}} ^+} {{\mathrm{K}} ^-}$ combinations in the $\phi {{\mathrm{K}} ^+} {{\mathrm{K}} ^-}$ and the $2 \times ({{\mathrm{K}} ^+} {{\mathrm{K}} ^-})$ components. The effect of a potential contribution from the $f_0 (980)$ state in the 2D fits is estimated by including the $f_0 (980)$ contribution following the analysis described in Ref. [44], and varying the $f_0 (980)$ mass and natural width within the uncertainties quoted in Ref. [14]. Contributions from multiple candidates are below 2% per event and are not considered in the evaluation of systematic uncertainties. The uncertainty related to the momentum-scale calibration is negligible.

The total systematic uncertainty is obtained as the quadratic sum of the individual systematic contributions. The systematic uncertainties are shown in Table 3. The description of the background and the potential contributions from other resonances dominate the total systematic uncertainties. In the yield ratios the systematic uncertainty is smaller than or comparable to the statistical uncertainty.

**Table 3:** Systematic uncertainties of the charmonium event yield ratios within families and with respect to the ${\eta _{{c}}} (1S)$ yield. The total uncertainty is the sum in quadrature of the individual contributions

Systematic uncertainty	$\frac{N_{{\upchi _{{{c}} 0}}}}{N_{{\eta _{{c}}} (1S)}}$	$\frac{N_{{\upchi _{{{c}} 1}}}}{N_{{\eta _{{c}}} (1S)}}$	$\frac{N_{{\upchi _{{{c}} 2}}}}{N_{{\eta _{{c}}} (1S)}}$	$\frac{N_{{\upchi _{{{c}} 1}}}}{N_{{\upchi _{{{c}} 0}}}}$	$\frac{N_{{\upchi _{{{c}} 2}}}}{N_{{\upchi _{{{c}} 0}}}}$	$\frac{N_{{\eta _{{c}}} (2S)}}{N_{{\eta _{{c}}} (1S)}}$
Including other states	0.004	0.003	0.003	0.006	0.008	0.003
Description of detector resolution	${<}0.001$	${<}0.001$	${<}0.001$	0.001	0.001	0.002
Description of signal resonances	0.002	0.002	0.001	0.010	0.002	0.003
Background model	0.010	0.005	0.005	0.002	0.012	${<}0.001$
2D fit functions	0.002	${<}0.001$	0.001	0.005	0.005	0.001
Total	0.011	0.006	0.006	0.012	0.015	0.005

Complementary cross-checks are performed by comparing the distributions of kinematic variables in simulation and data. The stability of the results is checked by using an alternative binning of the $\phi \phi$ invariant mass distribution (shifted by half a bin) and by using the weighted signal events from the *sPlot*  [45] instead of the nominal 2D fit technique. The same check is performed for the determination of the masses and widths of the charmonium states. In all cases no significant changes are observed and no additional contributions to the systematic uncertainties are assigned.

### Production of $$\varvec{{\eta _{{c}}}}$$ and $$\varvec{\chi _c}$$ in $${b} $$-hadron decays

The production ratios of charmonium *C* with respect to the ${\eta _{{c}}} (1S)$ yield and between pairs of $\upchi _{c}$ states

$$\begin{aligned} R^{C_1}_{C_2} \equiv \frac{{\mathcal {B}} ( {{b}} \rightarrow C_1\,X ) \times {\mathcal {B}} ( C_1 \rightarrow \phi \, \phi )}{{\mathcal {B}} ( {{b}} \rightarrow C_2\,X ) \times {\mathcal {B}} ( C_2 \rightarrow \phi \, \phi )} \end{aligned}$$

are determined to be

$$\begin{aligned} R^{{\upchi _{{{c}} 0}}}_{{\eta _{{c}}} (1S)}= & {} 0.147 \pm 0.023 \pm 0.011, \\ R^{{\upchi _{{{c}} 1}}}_{{\eta _{{c}}} (1S)}= & {} 0.073 \pm 0.016 \pm 0.006, \\ R^{{\upchi _{{{c}} 2}}}_{{\eta _{{c}}} (1S)}= & {} 0.081 \pm 0.013 \pm 0.005, \\ R^{{\upchi _{{{c}} 1}}}_{{\upchi _{{{c}} 0}}}= & {} 0.50 \pm 0.11 \pm 0.01, \\ R^{{\upchi _{{{c}} 2}}}_{{\upchi _{{{c}} 0}}}= & {} 0.56 \pm 0.10 \pm 0.01, \\ R^{{\eta _{{c}}} (2S)}_{{\eta _{{c}}} (1S)}= & {} 0.040 \pm 0.011 \pm 0.004, \end{aligned}$$

where, here and throughout, the first uncertainties are statistical and the second are systematic. The dominant contributions to the systematic uncertainty on the relative $\chi _c$ production rates arise from accounting for possible other resonances and from uncertainties on the $\chi _c$ masses [14]. The systematic uncertainties are smaller than the statistical uncertainties, so that the overall uncertainty on the measurements will be reduced with a larger dataset. The systematic uncertainty on the relative production rate of ${\eta _{{c}}} (2S)$ mesons is dominated by possible contributions from other resonances and variation of the ${\eta _{{c}}} (2S)$ natural width.

In the following, the $${\eta _{{c}}} (1S)$$ production rate in $${b} $$-hadron decays and the branching fractions of the charmonium decays to $$\phi \phi $$ are used to obtain single ratios of charmonium production rates in $${b} $$-hadron decays and the branching fractions of inclusive $${b} $$-hadron transitions to charmonium states. The $${\eta _{{c}}} (1S)$$ inclusive production rate in $${b} $$-hadron decays was measured by LHCb as $${\mathcal {B}} ( b \rightarrow {\eta _{{c}}} (1S) X ) = ( 4.88 \pm 0.97 ) \times 10^{-3}$$ [15] using decays to $${p} $$ $$\overline{{{p}}}$$. Branching fractions of the charmonia decays to $$\phi \phi $$ from Ref. [14] are used. However, Ref. [14] indicates a possible discrepancy for the $${\mathcal {B}} ( {\eta _{{c}}} (1S) \rightarrow \phi \phi )$$ value when comparing a direct determination and a fit including all available measurements. Therefore, an average of the results from Belle  [46] and BaBar  [47] using $${{{B}} ^+} $$ decays to $$\phi \phi {{\mathrm{K}} ^+} $$, $${\mathcal {B}} ( {\eta _{{c}}} (1S) \rightarrow \phi \phi ) = ( 3.21 \pm 0.72 ) \times 10^{-3}$$, is used below. The uncertainty of this average dominates the majority of the following results in this section, and an improved knowledge of the $${\mathcal {B}} ( {\eta _{{c}}} (1S) \rightarrow \phi \phi )$$ is critical to reduce the uncertainties of the derived results. The values $${\mathcal {B}} ( {\upchi _{{{c}} 0}} \rightarrow \phi \phi ) = (7.7 \pm 0.7) \times 10^{-4}$$, $${\mathcal {B}} ( {\upchi _{{{c}} 1}} \rightarrow \phi \phi ) = (4.2 \pm 0.5) \times 10^{-4}$$, and $${\mathcal {B}} ( {\upchi _{{{c}} 2}} \rightarrow \phi \phi ) = (1.12 \pm 0.10) \times 10^{-3}$$, are used for the $$\chi _c$$ decays [14]. The relative branching fractions of $${b} $$-hadron inclusive decays into $$\chi _c$$ states are then derived to be

$$\begin{aligned} \frac{{\mathcal {B}} ( b \rightarrow {\upchi _{{{c}} 1}} X )}{{\mathcal {B}} ( b \rightarrow {\upchi _{{{c}} 0}} X )}&= 0.92 \pm 0.20 \pm 0.02 \pm 0.14_{{\mathcal {B}}}, \\ \frac{{\mathcal {B}} ( b \rightarrow {\upchi _{{{c}} 2}} X )}{{\mathcal {B}} ( b \rightarrow {\upchi _{{{c}} 0}} X )}&= 0.38 \pm 0.07 \pm 0.01 \pm 0.05_{{\mathcal {B}}}, \end{aligned}$$

where the third uncertainty is due to those on the branching fractions $${\mathcal {B}} ( \chi _c \rightarrow \phi \phi )$$. The result for the relative $$\upchi _{{{c}} 1}$$ and $$\upchi _{{{c}} 2}$$ production in inclusive $${b} $$-hadron decays is close to the values measured in $${{B}} ^0$$ and $${{{B}} ^+} $$ production [14].

The branching fractions of $${b} $$-hadron decays into $$\chi _c$$ states relative to the $${\eta _{{c}}} (1S)$$ meson are

$$\begin{aligned} \frac{{\mathcal {B}} ( b \rightarrow {\upchi _{{{c}} 0}} X )}{{\mathcal {B}} ( b \rightarrow {\eta _{{c}}} (1S) X )}&= 0.62 \pm 0.10 \pm 0.05 \pm 0.15_{{\mathcal {B}}}, \\ \frac{{\mathcal {B}} ( b \rightarrow {\upchi _{{{c}} 1}} X )}{{\mathcal {B}} ( b \rightarrow {\eta _{{c}}} (1S) X )}&= 0.56 \pm 0.12 \pm 0.05 \pm 0.13_{{\mathcal {B}}}, \\ \frac{{\mathcal {B}} ( b \rightarrow {\upchi _{{{c}} 2}} X )}{{\mathcal {B}} ( b \rightarrow {\eta _{{c}}} (1S) X )}&= 0.23 \pm 0.04 \pm 0.02 \pm 0.06_{{\mathcal {B}}}, \end{aligned}$$

where the dominating uncertainty is due to the uncertainty of the branching fractions ${\mathcal {B}} ( {\eta _{{c}}} (1S) \rightarrow \phi \phi )$ and ${\mathcal {B}} ( \chi _c \rightarrow \phi \phi )$. The absolute branching fractions are determined to be

$$\begin{aligned} {\mathcal {B}} ( b \rightarrow {\upchi _{{{c}} 0}} X )&= ( 3.02 \pm 0.47 \pm 0.23 \pm 0.94_{{\mathcal {B}}} ) \times 10^{-3}, \\ {\mathcal {B}} ( b \rightarrow {\upchi _{{{c}} 1}} X )&= ( 2.76 \pm 0.59 \pm 0.23 \pm 0.89_{{\mathcal {B}}} ) \times 10^{-3}, \\ {\mathcal {B}} ( b \rightarrow {\upchi _{{{c}} 2}} X )&= ( 1.15 \pm 0.20 \pm 0.07 \pm 0.36_{{\mathcal {B}}} ) \times 10^{-3}, \end{aligned}$$

where the third uncertainty is due to the uncertainties on the branching fractions of the $${b} $$-hadron decays to the $${\eta _{{c}}} (1S)$$ meson, $${\mathcal {B}} ( {{b}} \rightarrow {\eta _{{c}}} (1S) X )$$, and of $${\eta _{{c}}} (1S)$$ and $$\chi _c$$ decays to $$\phi \phi $$. The branching fraction of $${b} $$-hadron decays into $$\upchi _{{{c}} 0}$$ is measured for the first time, and is found to be larger than the values predicted in Ref. [18].

Throughout the paper comparisons of the results on the production of charmonium states to theory predictions neglect the fact that the measured branching fractions also contain decays via intermediate higher-mass charmonium resonances, whereas theory calculations consider only direct $${b} $$-hadron transitions to the charmonium state considered. Among the contributions that can be quantified the most sizeable comes from the $$\uppsi {(2S)}$$ state decaying to the $$\chi _c$$ states. With the branching fraction $${\mathcal {B}} ( {{b}} \!\rightarrow {\uppsi {(2S)}} X )$$ recently measured [11] by LHCb the branching fractions $${\mathcal {B}} ( {{b}} \!\rightarrow \chi _c X )$$, measured in this paper, are influenced by about $$10 \%$$. The branching fractions $${\mathcal {B}} ( {{b}} \!\rightarrow {\upchi _{{{c}} 0}} X )$$ and $${\mathcal {B}} ( {{b}} \!\rightarrow {\upchi _{{{c}} 2}} X )$$ remain different from the predictions in Ref. [18].

The branching fraction measurement for $${b} $$-hadron decays into $$\upchi _{{{c}} 1}$$ is most precise in mixtures of $${{B}} ^0$$, $${{{B}} ^+} $$, $${{B}} ^0_{{s}} $$, $${{B}} _{{c}} ^+$$ and $${b} $$ baryons. The central value is lower than the central values measured by the DELPHI [8] and L3 [9] experiments at LEP, $$( 11.3 ^{+ 5.8} _{- 5.0} \pm 0.4 ) \times 10^{-3}$$ and $$( 19 \pm 7 \pm 1 ) \times 10^{-3}$$, respectively. For the measurements with different $${b} $$-hadron content, the LHCb result is consistent with measurements by CLEO  [2], Belle  [4], and BaBar  [5]. Finally, the LHCb result for the inclusive $${b} $$-hadron decays into $$\upchi _{{{c}} 1}$$ is consistent with the prediction in Ref. [18].

The branching fraction of $${b} $$-hadron decays into $$\upchi _{{{c}} 2}$$ is measured for the first time with a mixture of $${{B}} ^0$$, $${{{B}} ^+} $$, $${{B}} ^0_{{s}} $$, $${{B}} _{{c}} ^+$$ and $${b} $$-baryons. The result is consistent with the world average [14] measured with the $${{B}} ^0$$ and $${{{B}} ^+} $$ mixture, and with individual results from CLEO  [3], Belle  [4] and BaBar  [5]. The value obtained is below the range predicted in Ref. [18].

**Fig. 5:**
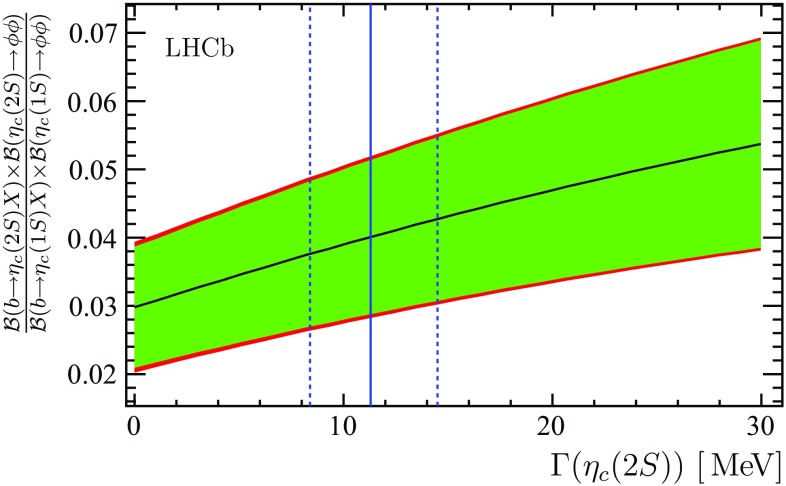
Ratio of the ${\eta _{{c}}} (2S)$ and ${\eta _{{c}}} (1S)$ inclusive yields $\frac{{\mathcal {B}} ( b \rightarrow {\eta _{{c}}} (2S) X ) \times {\mathcal {B}} ( {\eta _{{c}}} (2S) \rightarrow \phi \phi )}{{\mathcal {B}} ( b \rightarrow {\eta _{{c}}} (1S) X ) \times {\mathcal {B}} ( {\eta _{{c}}} (1S) \rightarrow \phi \phi )}$ as a function of the assumed ${\eta _{{c}}} (2S)$ natural width. Statistical (*green band*) and total uncertainties are shown separately. The ${\eta _{{c}}} (2S)$ natural width from Ref. [14] is shown as a *vertical solid line*; *the dashed lines* indicate its uncertainty

A deviation of the $${\eta _{{c}}} (2S)$$ natural width from the world average value [14] would affect the measured ratio of $${\eta _{{c}}} (2S)$$ and $${\eta _{{c}}} (1S)$$ production rates in $${b} $$-hadron inclusive decays, as shown in Fig. 5. The decay $${\eta _{{c}}} (2S) \rightarrow \phi \phi $$ has not been observed so far. Hence the product of the branching fraction of $${b} $$-hadron decays to $${\eta _{{c}}} (2S)$$ and the branching fraction of the $${\eta _{{c}}} (2S) \rightarrow \phi \phi $$ decay mode is determined as

$$\begin{aligned}&{\mathcal {B}} ( b \rightarrow {\eta _{{c}}} (2S) X ) \times {\mathcal {B}} ( {\eta _{{c}}} (2S) \rightarrow \phi \phi )\\&\quad = ( 6.34 \pm 1.81 \pm 0.57 \pm 1.89 ) \times 10^{-7}, \end{aligned}$$

where the systematic uncertainty is dominated by the uncertainty on the $${\eta _{{c}}} (1S)$$ production rate in $${b} $$-hadron decays. This is the first evidence for $${\eta _{{c}}} (2S)$$ production in $${b} $$-hadron decays, and for the decay of the $${\eta _{{c}}} (2S)$$ meson into a pair of $$\phi $$ mesons.

### Transverse momentum dependence of the differential cross-sections for $\varvec{{\eta _{{c}}} (1S)}$ and $\varvec{\chi _c}$ production

**Fig. 6:**
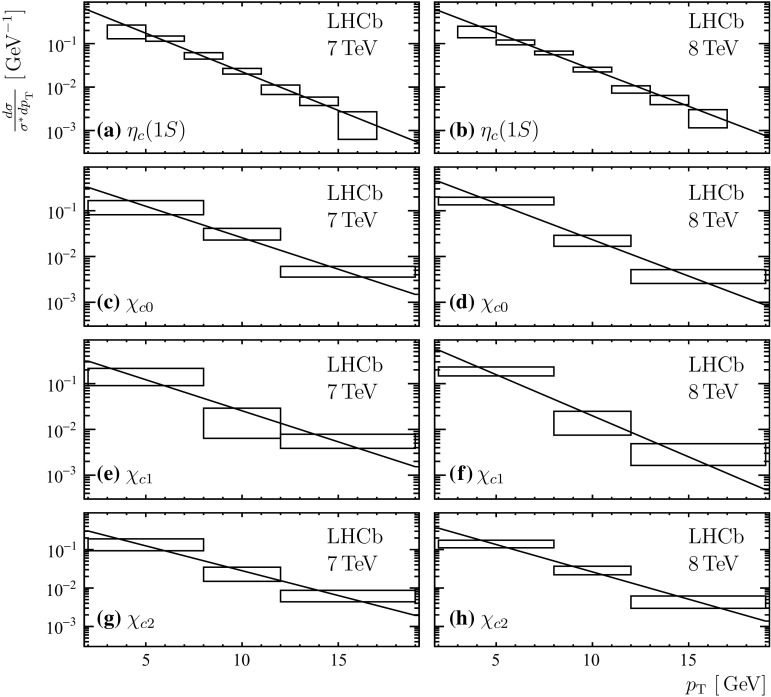
Differential cross-sections normalized to the production cross-section integrated over the studied region, $\sigma ^*$, of the (*top to bottom*) ${\eta _{{c}}} (1S)$, $\upchi _{{{c}} 0}$, $\upchi _{{{c}} 1}$ and $\upchi _{{{c}} 2}$ states for the (*left*) $\sqrt{s} = 7 {~\mathrm{TeV}}$ and the (*right*) $\sqrt{s} = 8 {~\mathrm{TeV}}$ data samples. The *horizontal* and *vertical size of the boxes* reflect the size of the $p_{\mathrm{T}}$ bins and the statistical and uncorrelated systematic uncertainties of the differential production cross-sections added in quadrature. The exponential functions proportional to $\exp (- \alpha \, p_{\mathrm{T}})$ fitted to the integral of the each bin of the distributions are overlaid

The shapes of the differential production cross-sections as a function of transverse momentum are studied in the LHCb acceptance ($$2< \eta < 5$$) and for $$3< p_{\mathrm{T}} < 17 {\,\mathrm{GeV}} $$ and $$2< p_{\mathrm{T}} < 19 {\,\mathrm{GeV}} $$ for the $${\eta _{{c}}} (1S)$$ and $$\chi _c$$ states, respectively. Each differential production cross-section is normalized to the production cross-section integrated over the studied $$p_{\mathrm{T}}$$ region. Figure 6 shows the normalized differential cross-sections of $${\eta _{{c}}} (1S)$$, $$\upchi _{{{c}} 0}$$, $$\upchi _{{{c}} 1}$$ and $$\upchi _{{{c}} 2}$$ production at $$\sqrt{s} = 7$$ and $$8 {~\mathrm{TeV}}$$. An exponential function proportional to $$\exp (- \alpha \, p_{\mathrm{T}})$$ is fitted to the integral of the each bin of the distributions. No significant difference is observed between the $$\sqrt{s} = 7 {~\mathrm{TeV}}$$ and $$8 {~\mathrm{TeV}}$$ data. The results for the slope parameters $$\alpha $$ are given in Table 4. For $$\upchi _{{{c}} 1}$$ and $$\upchi _{{{c}} 2}$$ production in $${b} $$-hadron decays these results extend the ATLAS studies [48] in $$p_{\mathrm{T}}$$ and rapidity.

**Table 4:** Exponential slope parameter in units of ${\,\mathrm{GeV}} ^{-1}$ from a fit to the $p_{\mathrm{T}}$ spectra of ${\eta _{{c}}} (1S)$, $\upchi _{{{c}} 0}$, $\upchi _{{{c}} 1}$ and $\upchi _{{{c}} 2}$ mesons

	${\eta _{{c}}} (1S)$	$\upchi _{{{c}} 0}$	$\upchi _{{{c}} 1}$	$\upchi _{{{c}} 2}$
$\sqrt{s} = 7 {~\mathrm{TeV}}$	$0.41 \pm 0.02$	$0.32 \pm 0.04$	$0.31 \pm 0.06$	$0.30 \pm 0.05$
$\sqrt{s} = 8 {~\mathrm{TeV}}$	$0.39 \pm 0.02$	$0.37 \pm 0.04$	$0.41 \pm 0.06$	$0.33 \pm 0.04$

### Search for production of $\varvec{X(3872)}$, $\varvec{X(3915)}$ and $\varvec{{\upchi _{{{c}} 2}} (2P)}$

The observation of the *X*(3915) and $${\upchi _{{{c}} 2}} (2P)$$ states in $${b} $$-hadron decays or the *X*(3872) decaying to a pair of $$\phi $$ mesons would provide interesting information on the properties of these states. The invariant mass spectrum of $$\phi \phi $$ combinations in Fig. 4 shows no evidence for a signal from the *X*(3872), *X*(3915), or $${\upchi _{{{c}} 2}} (2P)$$ states. Bayesian upper limits assuming a uniform prior in the event yields are obtained on the branching fractions relative to those involving decays to the states with similar quantum numbers. For the states with similar quantum numbers, in the efficiency ratios systematic uncertainties largely cancel. Using the efficiency ratios from the simulation, the upper limits at $$95 \%$$ ($$90 \%$$) CL on the ratios of inclusive branching fractions are

$$\begin{aligned} R^{X(3872)}_{{\upchi _{{{c}} 1}}}< & {} 0.39 \ (0.34), \\ R^{X(3915)}_{{\upchi _{{{c}} 0}}}< & {} 0.14 \ (0.12), \\ R^{{\upchi _{{{c}} 2}} (2P)}_{{\upchi _{{{c}} 2}}}< & {} 0.20 \ (0.16). \end{aligned}$$

Using the measured production rates of the $$\chi _c$$ states in $${b} $$-hadron decays and branching fractions for the $$\chi _c$$ decays to the $$\phi \phi $$ final state [14], the upper limits at $$95\%$$ ($$90 \%$$) CL on the production rates of the *X*(3872), *X*(3915), and $${\upchi _{{{c}} 2}} (2P)$$ states in $${b} $$-hadron decays are

$$\begin{aligned} {\mathcal {B}} ( {{b}} \rightarrow X(3872) X ) \times {\mathcal {B}} ( X(3872) \rightarrow \phi \phi )< & {} 4.5 \ (3.9) \times 10^{-7}, \\ {\mathcal {B}} ( {{b}} \rightarrow X(3915) X ) \times {\mathcal {B}} ( X(3915) \rightarrow \phi \phi )< & {} 3.1 \ (2.7) \times 10^{-7}, \\ {\mathcal {B}} ( {{b}} \rightarrow {\upchi _{{{c}} 2}} (2P) X ) \times {\mathcal {B}} ( {\upchi _{{{c}} 2}} (2P) \rightarrow \phi \phi )< & {} 2.8 \ (2.3) \times 10^{-7}. \end{aligned}$$

## Masses and natural widths of charmonium states

The majority of the $${\eta _{{c}}} (1S)$$ mass measurements, used in the fit of Ref. [14], were performed with two-photon production, $$\gamma \gamma \rightarrow {\eta _{{c}}} (1S) \rightarrow \text{ hadrons }$$, radiative decays $${{{J}/\uppsi }} \rightarrow {\eta _{{c}}} (1S) \gamma $$ and $${\uppsi {(2S)}} \rightarrow {\eta _{{c}}} (1S) \gamma $$, $${{p}} {\overline{{{p}}}} \rightarrow {\eta _{{c}}} (1S) \rightarrow \gamma \gamma $$, and exclusive $${B} $$ decays, yielding the average value $$2983.4 \pm 0.5 {\,\mathrm{MeV}} $$. Mass determinations via exclusive *B* decays, performed at the BaBar and Belle experiments [49–51], do not provide consistent results. In 2009, the CLEO collaboration observed a significant asymmetry in the line shapes of radiative $${{{J}/\uppsi }} \rightarrow \gamma {\eta _{{c}}} (1S)$$ and $${\uppsi {(2S)}} \rightarrow \gamma {\eta _{{c}}} (1S)$$ transitions [52], which, when ignored, could lead to significant bias in the mass and width measurement via $${{J}/\uppsi }$$ or $$\uppsi {(2S)}$$ radiative decays. Recent BES III results [53, 54] obtained using radiative decays of $$\uppsi {(2S)}$$, shifted the world average value by more than two standard deviations. Therefore precise $${\eta _{{c}}} (1S)$$ mass measurements using a different technique are needed. LHCb measured $$M_{{\eta _{{c}}} (1S)} = 2982.2 \pm 1.5 \pm 0.1 {\,\mathrm{MeV}} $$ [15] using $${\eta _{{c}}} (1S)$$ from $${b} $$-hadron decays and reconstructing $${\eta _{{c}}} (1S)$$ via decays to $${{p}} {\overline{{{p}}}} $$. A similar situation occurs with the $${\eta _{{c}}} (1S)$$ natural width determination, where recent BES III results obtained using radiative decays of $$\uppsi {(2S)}$$ shifted the world average from $$29.7 \pm 1.0 {\,\mathrm{MeV}} $$ to $$31.8 \pm 0.8 {\,\mathrm{MeV}} $$.

The properties of the $${\eta _{{c}}} (2S)$$ state are less well studied. Measurements at the CLEO  [55], BaBar  [56, 57], Belle  [51, 58] and BES III [59, 60] experiments, using $$\gamma \gamma \rightarrow {\eta _{{c}}} (2S) \rightarrow \text{ hadrons }$$, double charmonium production in $${e} ^+{e} ^-$$ annihilation, exclusive $${B} $$ decays and radiative transitions of $$\uppsi {(2S)}$$, yield the world averages [14] of $$3639.4 \pm 1.3 {\,\mathrm{MeV}} $$ for the $${\eta _{{c}}} (2S)$$ mass, and $$11.3 ^{+ 3.2} _{-2.9} {\,\mathrm{MeV}} $$ for its natural width.

Table 5 presents measurements of the masses of the ${\eta _{{c}}}$ and $\chi _c$ states and of the natural width of the ${\eta _{{c}}} (1S)$ from the fit of the $\phi \phi$ invariant mass spectrum in Fig. 4. For the determination of the systematic uncertainties, except for the test of the impact of the $f_0 (980)$ meson, the same variations of the analysis are performed as for the determination of the charmonium yields. In addition, the effect of excluding the ${\eta _{{c}}} (2S)$ mass region ($2.8 {-} 3.7 {\,\mathrm{GeV}}$) is studied, and the uncertainties related to the momentum-scale calibration are estimated by varying the calibration parameter by ${\pm } 3 \times 10^{-4}$ [33]. The resulting total systematic uncertainty is obtained as the quadratic sum of the individual contributions. The uncertainty related to the momentum-scale calibration dominates the mass determination for all ${\eta _{{c}}}$ and $\chi _c$ states. The uncertainty of the $\Gamma _{{\eta _{{c}}} (1S)}$ measurement is dominated by the background description.

**Table 5:** Charmonium masses and natural widths in ${\,\mathrm{MeV}}$

	Measured value	World average [14]
$M_{{\eta _{{c}}} (1S)}$	$2982.8 \pm 1.0 \pm 0.5$	$2983.4 \pm 0.5$
$M_{{\upchi _{{{c}} 0}}}$	$3413.0 \pm 1.9 \pm 0.6$	$3414.75 \pm 0.31$
$M_{{\upchi _{{{c}} 1}}}$	$3508.4 \pm 1.9 \pm 0.7$	$3510.66 \pm 0.07$
$M_{{\upchi _{{{c}} 2}}}$	$3557.3 \pm 1.7 \pm 0.7$	$3556.20 \pm 0.09$
$M_{{\eta _{{c}}} (2S)}$	$3636.4 \pm 4.1 \pm 0.7$	$3639.2 \pm 1.2$
$\Gamma _{{\eta _{{c}}} (1S)}$	$31.4 \pm 3.5 \pm 2.0$	$31.8 \pm 0.8$
$\Gamma _{{\eta _{{c}}} (2S)}$	–	$$11.3^{\, \ + \, \ 3.2}_{\, \ - \, \ 2.9}\ $$

The measured charmonium masses agree with the world averages [14]. The measured ${\eta _{{c}}} (1S)$ mass is in agreement with the previous LHCb measurement using decays to the ${{p}} {\overline{{{p}}}}$ final states [15] and has a better precision. The precision obtained for the ${\eta _{{c}}} (1S)$ mass is comparable to the precision of the world average value. The value of the ${\eta _{{c}}} (1S)$ natural width is consistent with the world average [14].

**Table 6:** Charmonium mass differences (in ${\,\mathrm{MeV}}$)

	Measured value	World average [14]
$M_{{\upchi _{{{c}} 1}}} - M_{{\upchi _{{{c}} 0}}}$	$95.4 \pm 2.7 \pm 0.1$	$95.91 \pm 0.83$
$M_{{\upchi _{{{c}} 2}}} - M_{{\upchi _{{{c}} 0}}}$	$144.3 \pm 2.6 \pm 0.2$	$141.45 \pm 0.32$
$M_{{\eta _{{c}}} (2S)} - M_{{\eta _{{c}}} (1S)}$	$653.5 \pm 4.2 \pm 0.4$	$655.70 \pm 1.48$

The charmonium mass differences $M_{{\upchi _{{{c}} 1}}} - M_{{\upchi _{{{c}} 0}}}$, $M_{{\upchi _{{{c}} 2}}} - M_{{\upchi _{{{c}} 0}}}$, and $M_{{\eta _{{c}}} (2S)} - M_{{\eta _{{c}}} (1S)}$ are obtained (Table 6) as a consistency check and for comparison with theory. For the determination of the systematic uncertainties the same variations of the analysis are performed as for the determination of the charmonium masses and widths. The uncertainty related to the 2D fit dominates the $M_{{\upchi _{{{c}} 1}}} - M_{{\upchi _{{{c}} 0}}}$ mass difference measurement. The systematic uncertainty of the $M_{{\upchi _{{{c}} 2}}} - M_{{\upchi _{{{c}} 0}}}$ measurement is dominated by the uncertainty related to potential contributions from other resonances and by the uncertainty on the background model. The uncertainty related to the momentum-scale calibration dominates the $M_{{\eta _{{c}}} (2S)} - M_{{\eta _{{c}}} (1S)}$ mass difference measurement. The measured charmonium mass differences agree with the world averages.

**Fig. 7:**
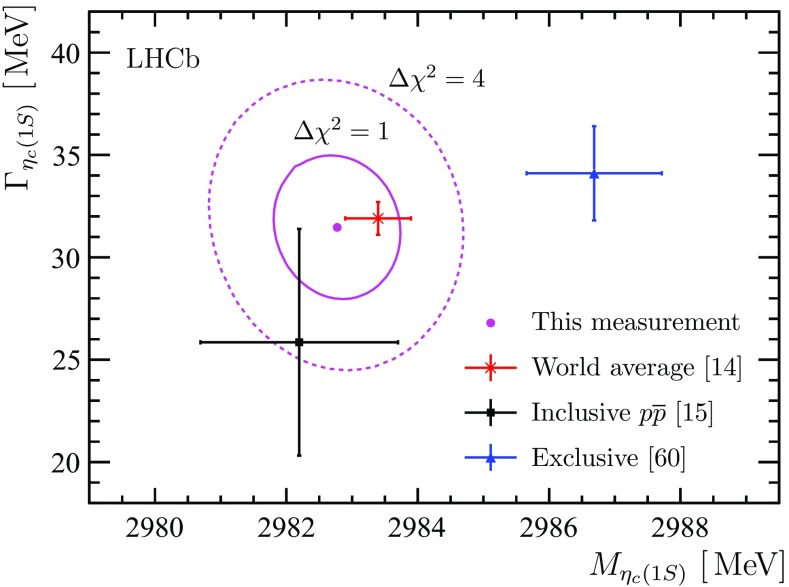
Contour plot of $\Gamma _{{\eta _{{c}}} (1S)}$ and $M_{{\eta _{{c}}} (1S)}$ using ${\eta _{{c}}} \rightarrow \phi \phi$ decays. The *two magenta curves* indicate $\Delta \chi ^2 = 1$ and $\Delta \chi ^2 = 4$ contours. Only statistical uncertainties are shown. The *red cross*, *black square* and *blue triangle with error bars* indicate the world average [14], the result from Ref. [15], and the result from Ref. [61], respectively

Figure 7 shows the $$\Gamma _{{\eta _{{c}}} (1S)}, \, M_{{\eta _{{c}}} (1S)}$$ contour plot, obtained from the analysis of $${b} $$-hadron decays into $$\eta _{{c}} $$ mesons, where the $$\eta _{{c}} $$ candidates are reconstructed via the decay $${\eta _{{c}}} (1S) \rightarrow \phi \phi $$. The measurements of the $${\eta _{{c}}} (1S)$$ mass and natural width using $${\eta _{{c}}} (1S)$$ meson decays to $$\phi \phi $$ are consistent with the studies using decays to $${p} $$ $$\overline{{{p}}}$$  [15] and with the world average [14]. The measured $${\eta _{{c}}} (1S)$$ mass is below the result in Ref. [61]. The precision obtained on the $${\eta _{{c}}} (1S)$$ mass is comparable to the precision of the world average.

## First evidence of the $\varvec{{{{B}} ^0_{{s}}} \rightarrow \phi \phi \phi }$ decay

**Fig. 8:**
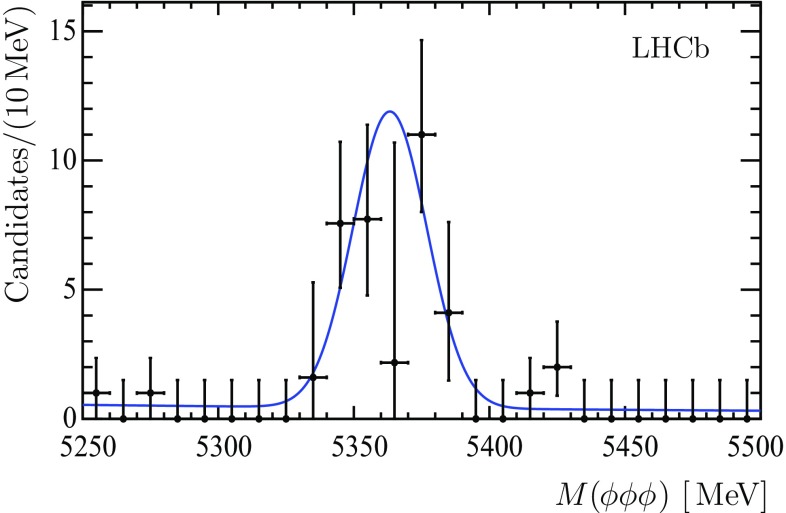
Invariant mass spectrum of the $$\phi \phi \phi $$ combinations in the region of the $${{B}} ^0_{{s}} $$ mass, including the fit function described in the text

In order to extract $$\phi \phi \phi $$ combinations a 3D extended unbinned maximum likelihood fit is used, as described in Sect. 3. Figure 8 shows the invariant mass distribution for $$\phi \phi \phi $$ combinations. The fit to the invariant $$\phi \phi \phi $$ mass spectrum is performed using a sum of two Gaussian functions with a common mean to describe the $${{B}} ^0_{{s}} $$ signal, and an exponential function to describe combinatorial background. The ratio of the two Gaussian widths and the fraction of the narrow Gaussian are taken from simulation so that a single free parameter in the $$\phi \phi \phi $$ invariant mass fit accounts for the detector resolution. A signal of $$41 \pm 10 \pm 5$$ $${{B}} ^0_{{s}} $$ decays over a low background of about 3 events is obtained. Uncertainties related to the background description in the 3D fit and to the decay model defining the $$\phi $$ polarization dominate the systematic uncertainty in the $${{B}} ^0_{{s}} $$ signal yield determination. The significance of the signal is estimated from the distributions of the difference in the logarithm of the best-fit $$\chi ^2$$ with and without including the signal shape in toy simulation samples. This leads to a signal significance of 4.9 standard deviations.

**Fig. 9:**
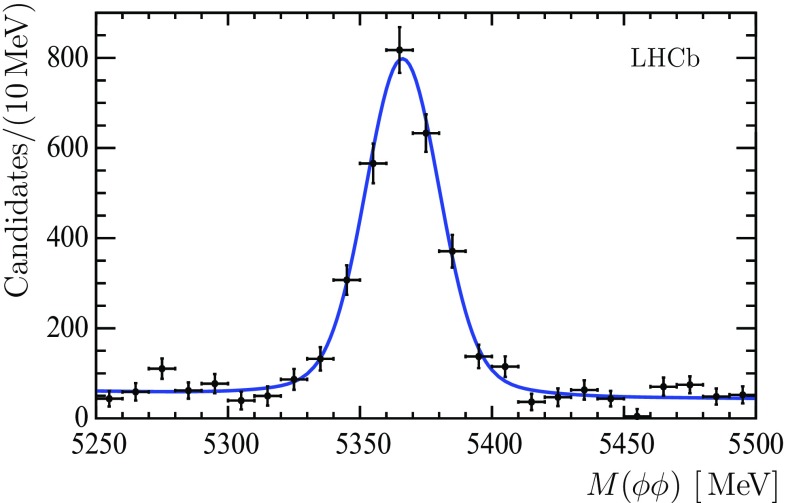
Invariant mass spectrum of the $$\phi \phi $$ combinations in the region of the $${{B}} ^0_{{s}} $$ mass, including the fit function described in the text

The $${{{B}} ^0_{{s}}} \rightarrow \phi \phi $$ decay mode is chosen as a normalization mode for the $${\mathcal {B}} ( {{{B}} ^0_{{s}}} \rightarrow \phi \phi \phi )$$ measurement. The invariant mass spectrum obtained from 2D fits in bins of the $$\phi \phi $$ invariant mass in the region of the $${{B}} ^0_{{s}} $$ mass is shown in Fig. 9. A sum of two Gaussian functions with a common mean is used to describe the $${{B}} ^0_{{s}} $$ signal shape, while an exponential function models the combinatorial background. The ratio of the two Gaussian widths and the fraction of the narrow Gaussian function are taken from simulation. In total $$2701 \pm 114 \pm 84$$ $${{B}} ^0_{{s}} $$ decays are found. The uncertainties related to the description of the resolution in the 2D fits and the description of the $$\phi \phi $$ invariant mass resolution dominate the systematic uncertainty in the $${{B}} ^0_{{s}} $$ signal yield determination.

The ratio of the ${{{B}} ^0_{{s}}} \rightarrow \phi \phi \phi$ and ${{{B}} ^0_{{s}}} \rightarrow \phi \phi$ branching fractions is obtained from the relative ${{{B}} ^0_{{s}}} \rightarrow \phi \phi \phi$ and ${{{B}} ^0_{{s}}} \rightarrow \phi \phi$ signal yields and their efficiencies as

$$\begin{aligned} \frac{{\mathcal {B}} ( {{{B}} ^0_{{s}}} \rightarrow \phi \phi \phi )}{{\mathcal {B}} ( {{{B}} ^0_{{s}}} \rightarrow \phi \phi )}= & {} \frac{N_{{{{B}} ^0_{{s}}} \rightarrow \phi \phi \phi }}{N_{{{{B}} ^0_{{s}}} \rightarrow \phi \phi }} \times \frac{\varepsilon _{{{{B}} ^0_{{s}}} \rightarrow \phi \phi }}{\varepsilon _{{{{B}} ^0_{{s}}} \rightarrow \phi \phi \phi }} \times \frac{1}{{\mathcal {B}} ( \phi \rightarrow {{\mathrm{K}} ^+} {{\mathrm{K}} ^-})}\\ =& {} 0.117 \pm 0.030 \pm 0.015. \end{aligned}$$

In the above expression, the event yields are determined from the fits. The efficiency ratio, $$\varepsilon _{{{{B}} ^0_{{s}}} \rightarrow \phi \phi \phi } / \varepsilon _{{{{B}} ^0_{{s}}} \rightarrow \phi \phi } = 0.26 \pm 0.01$$, is obtained from simulation and corrected to account for different $${{B}} ^0_{{s}} $$ transverse momentum spectra in data and simulation. The $${{{B}} ^0_{{s}}} \rightarrow \phi \phi \phi $$ transition is assumed to proceed via a three-body decay with uniform phase-space density. This assumption is supported below by comparing the $$\phi \phi $$ invariant mass distribution in data and simulation. The systematic uncertainty is dominated by the uncertainty in polarization of the $$\phi $$ mesons in the decay $${{{B}} ^0_{{s}}} \rightarrow \phi \phi \phi $$, as discussed at the end of this section. Using the branching fraction of the $${{{B}} ^0_{{s}}} \rightarrow \phi \phi $$ decay, $${\mathcal {B}} ( {{{B}} ^0_{{s}}} \rightarrow \phi \phi ) = (1.84 \pm 0.05 \pm 0.07 \pm 0.11_{f_s / f_d} \pm 0.12_{\mathrm{norm}}) \times 10^{-5}$$ [22], the branching fraction for the $${{B}} ^0_{{s}} $$ meson decay to three $$\phi $$ mesons is determined to be

$$\begin{aligned} {\mathcal {B}} ( {{{B}} ^0_{{s}}} \rightarrow \phi \phi \phi ) = ( 2.15 \pm 0.54 \pm 0.28 \pm 0.21_{{\mathcal {B}}} ) \times 10^{-6}, \end{aligned}$$

where the last uncertainty is due to the branching fraction ${\mathcal {B}} ( {{{B}} ^0_{{s}}} \rightarrow \phi \phi )$.

**Fig. 10:**
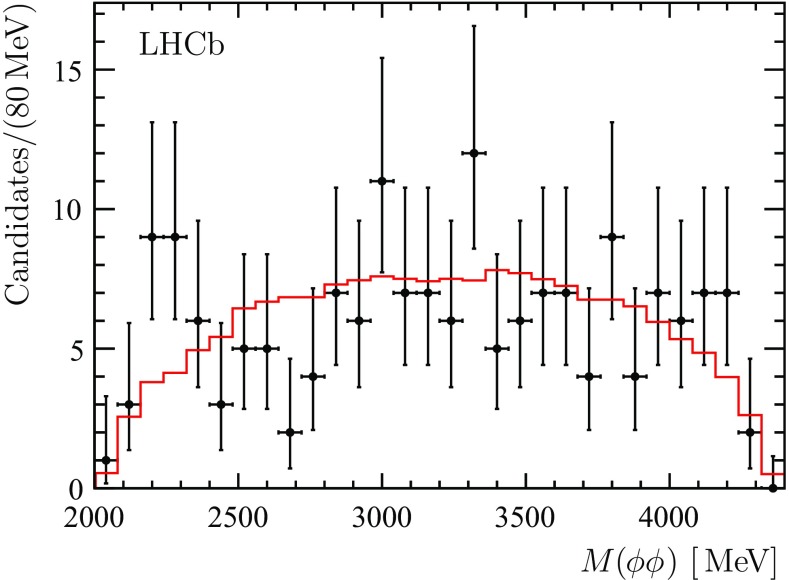
The invariant mass distribution of each combination of $$\phi \phi $$ pairs in the $${{{B}} ^0_{{s}}} \rightarrow \phi \phi \phi $$ candidates. The $${{B}} ^0_{{s}} $$ candidates are constrained to the known $${{B}} ^0_{{s}} $$ mass. A phase-space distribution as obtained from simulation (*red histogram*) is overlaid

The $${{{B}} ^0_{{s}}} \rightarrow \phi \phi \phi $$ transition can proceed via a two-body decay involving intermediate resonances or via a three-body $${{{B}} ^0_{{s}}} \rightarrow \phi \phi \phi $$ decay. In order to search for contributions from possible intermediate resonances, the invariant mass of each $$\phi \phi $$ combination from all $${{{B}} ^0_{{s}}} \rightarrow \phi \phi \phi $$ candidates in the signal region of $$\pm 3$$ standard deviations around the $${{B}} ^0_{{s}} $$ mass is examined, see Fig. 10. The $${{B}} ^0_{{s}} $$ candidates are constrained to the known $${{B}} ^0_{{s}} $$ mass. Three entries to the histogram are produced by each $${{B}} ^0_{{s}} $$ candidate. A phase-space distribution as obtained from simulation is overlaid for comparison. No indication of significant contributions from $$\eta _{{c}} $$, $${\upchi _{c}} $$, $$f_2 (2300)$$ or $$f_2 (2340)$$ states is seen. A symmetrized Dalitz plot constructed following the approach described in Ref. [62] shows no evidence for resonant contributions either.

**Fig. 11:**
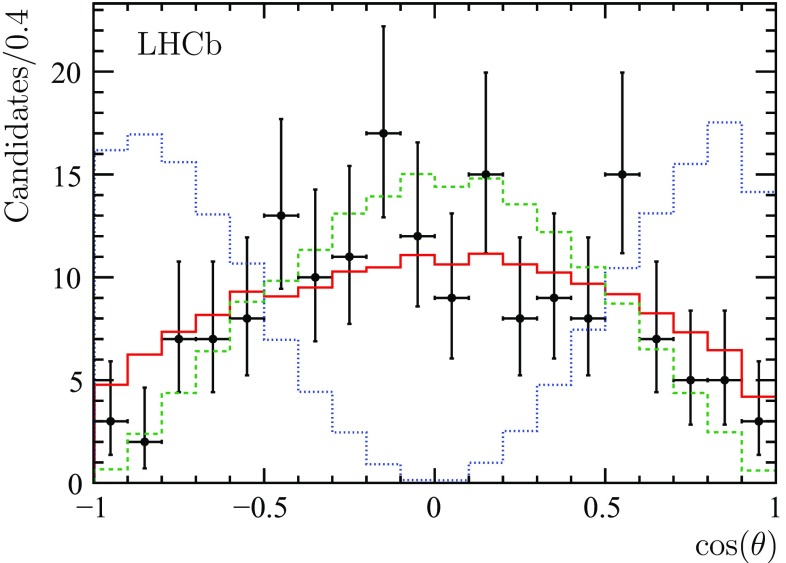
The $\phi$ meson angular distribution for the ${{{B}} ^0_{{s}}} \rightarrow \phi \phi \phi$ candidates (*points with error bars*) with the overlaid distribution from the simulation with no polarization (*red solid histogram*) and two extreme, transverse (*green dashed histogram*) and longitudinal (*blue dotted histogram*), polarizations

The polarization of the $$\phi $$ mesons is studied by means of the angle $$\theta $$ between the direction of flight of a $$\phi $$ meson in the $${{B}} ^0_{{s}} $$ rest frame and the $${{B}} ^0_{{s}} $$ direction in the laboratory frame. With the limited sample of $${{{B}} ^0_{{s}}} \rightarrow \phi \phi \phi $$ candidates the 3D fit technique to remove contributions from $${{\mathrm{K}} ^+} {{\mathrm{K}} ^-} $$ combinations that are not from $$\phi $$ decays cannot be used for this measurement. Instead, all $$\phi $$ mesons contributing in the mass range of the $${{B}} ^0_{{s}} $$ are used, with an estimated signal purity of $$71 \%$$. Figure 11 compares the $$\cos ( \theta )$$ distribution for the $${{{B}} ^0_{{s}}} \rightarrow \phi \phi \phi $$ signal candidates in data with expectations from simulation using different assumptions for the polarization. The purely longitudinal polarization clearly does not describe the data. The difference between the expectations for no polarization and purely transverse polarization is used to estimate the corresponding systematic uncertainty in the $${\mathcal {B}} ( {{{B}} ^0_{{s}}} \rightarrow \phi \phi \phi )$$ measurement. The most probable value for the fraction of transverse polarization, $$f_{\text {T}}$$, is found to be $$f_{\text {T}} = 0.86$$. Assuming a uniform prior in the physically allowed range, a Bayesian lower limit of $$f_{\text {T}} > 0.28$$ at $$95\%$$ CL is found.

## Summary and discussion

Charmonium production in $${b} $$-hadron inclusive decays is studied in $${p} $$ $${p} $$ collisions collected at $$\sqrt{s} = 7$$ and $$8 {~\mathrm{TeV}}$$ corresponding to an integrated luminosity of $$3.0 {\,\mathrm{fb}}^{-1} $$, using charmonium decays to $$\phi $$-meson pairs. The masses and natural widths of the $$\eta _{{c}} $$ and $$\chi _c$$ states are determined. In addition, the first evidence of $${{{B}} ^0_{{s}}} \rightarrow \phi \phi \phi $$ decay is obtained.

Ratios of charmonium *C* production rates,

$$\begin{aligned} R^{C_1}_{C_2} \equiv \frac{{\mathcal {B}} ( {{b}} \rightarrow C_1\,X ) \times {\mathcal {B}} ( C_1 \rightarrow \phi \, \phi )}{{\mathcal {B}} ( {{b}} \rightarrow C_2\,X ) \times {\mathcal {B}} ( C_2 \rightarrow \phi \, \phi )}, \end{aligned}$$

are measured to be

$$\begin{aligned} R^{{\upchi _{{{c}} 0}}}_{{\eta _{{c}}} (1S)}= & {} 0.147 \pm 0.023 \pm 0.011, \\ R^{{\upchi _{{{c}} 1}}}_{{\eta _{{c}}} (1S)}= & {} 0.073 \pm 0.016 \pm 0.006, \\ R^{{\upchi _{{{c}} 2}}}_{{\eta _{{c}}} (1S)}= & {} 0.081 \pm 0.013 \pm 0.005, \\ R^{{\upchi _{{{c}} 1}}}_{{\upchi _{{{c}} 0}}}= & {} 0.50 \pm 0.11 \pm 0.01, \\ R^{{\upchi _{{{c}} 2}}}_{{\upchi _{{{c}} 0}}}= & {} 0.56 \pm 0.10 \pm 0.01, \\ R^{{\eta _{{c}}} (2S)}_{{\eta _{{c}}} (1S)}= & {} 0.040 \pm 0.011 \pm 0.004, \end{aligned}$$

where the first uncertainties are statistical and the second ones are systematic. Using the branching fractions of $$\chi _c$$ decays to $$\phi \phi $$ from Ref. [14], relative branching fractions of $${b} $$ hadrons decaying inclusively to $$\chi _c$$ states are derived,

$$\begin{aligned} \frac{{\mathcal {B}} ( b \rightarrow {\upchi _{{{c}} 1}} X )}{{\mathcal {B}} ( b \rightarrow {\upchi _{{{c}} 0}} X )}= & {} 0.92 \pm 0.20 \pm 0.02 \pm 0.14_{{\mathcal {B}}}, \\ \frac{{\mathcal {B}} ( b \rightarrow {\upchi _{{{c}} 2}} X )}{{\mathcal {B}} ( b \rightarrow {\upchi _{{{c}} 0}} X )}= & {} 0.38 \pm 0.07 \pm 0.01 \pm 0.05_{{\mathcal {B}}}, \end{aligned}$$

where the third uncertainty is due to the branching fractions $${\mathcal {B}} ( \chi _c \rightarrow \phi \phi )$$. These results are consistent with the ratio of the $$\upchi _{{{c}} 1}$$ and $$\upchi _{{{c}} 2}$$ production rates measured in $${{B}} ^0$$ and $${{{B}} ^+} $$ decays [14].

Inclusive production rates of the $$\chi _c$$ states in $${b} $$-hadron decays are derived using branching fractions of the $$\chi _c$$ decays to $$\phi \phi $$ from Ref. [14], an average of the results from Belle  [46] and BaBar  [47] $${\mathcal {B}} ( {\eta _{{c}}} (1S) \rightarrow \phi \phi ) = ( 3.21 \pm 0.72 ) \times 10^{-3}$$, and the $${\eta _{{c}}} (1S)$$ inclusive production rate measured using decays to $${p} $$ $$\overline{{{p}}}$$, $${\mathcal {B}} ( b \rightarrow {\eta _{{c}}} (1S) X ) = ( 4.88 \pm 0.97 ) \times 10^{-3}$$ [15]. They are

$$\begin{aligned} {\mathcal {B}} ( b \rightarrow {\upchi _{{{c}} 0}} X )= & {} ( 3.02 \pm 0.47 \pm 0.23 \pm 0.94_{{\mathcal {B}}} ) \times 10^{-3}, \\ {\mathcal {B}} ( b \rightarrow {\upchi _{{{c}} 1}} X )= & {} ( 2.76 \pm 0.59 \pm 0.23 \pm 0.89_{{\mathcal {B}}} ) \times 10^{-3}, \\ {\mathcal {B}} ( b \rightarrow {\upchi _{{{c}} 2}} X )= & {} ( 1.15 \pm 0.20 \pm 0.07 \pm 0.36_{{\mathcal {B}}} ) \times 10^{-3}, \end{aligned}$$

where the third uncertainty is due to the uncertainties on the branching fraction of the $${b} $$-hadron decays to the $${\eta _{{c}}} (1S)$$ meson, $${\mathcal {B}} ( {{b}} \rightarrow {\eta _{{c}}} (1S) X )$$, and $${\eta _{{c}}} (1S)$$ and $$\chi _c$$ decays to $$\phi \phi $$. No indirect contribution to the production rate is subtracted. However, since contributions from $$\uppsi {(2S)}$$ decays to the $$\chi _c$$ states are limited, the results disfavour dominance of either colour-octet or colour-singlet contributions. The observed relations between the $$\chi _c$$ branching fractions are not consistent with those predicted in Ref. [18]. The branching fraction $${\mathcal {B}} ( b \rightarrow {\upchi _{{{c}} 0}} X )$$ is measured for the first time. The result for $${b} $$-hadron decays into $$\upchi _{{{c}} 1}$$ is the most precise measurement for the mixture of $${{B}} ^0$$, $${{{B}} ^+} $$, $${{B}} ^0_{{s}} $$, $${{B}} _{{c}} ^+$$ and $${b} $$-baryons. The central value of the result for $${b} $$-hadron decays into $$\upchi _{{{c}} 1}$$ is lower than the central values measured by the DELPHI [8] and L3 [9] experiments at LEP. The value obtained is consistent with the branching fraction of $${b} $$-hadron decays into $$\upchi _{{{c}} 1}$$ measured by CLEO  [2], Belle  [4] and BaBar  [5] with the light mixture of $${{B}} ^0$$ and $${{{B}} ^+} $$. The branching fraction of $${b} $$-hadron decays into $$\upchi _{{{c}} 2}$$ is measured for the first time with the $${{B}} ^0$$, $${{{B}} ^+} $$, $${{B}} ^0_{{s}} $$ and $${b} $$-baryons mixture. The result is consistent with the world average corresponding to the $${{B}} ^0$$, $${{{B}} ^+} $$ mixture [14] and with individual measurements from CLEO  [3], Belle  [4], and BaBar  [5].

Scaled differential charmonium production cross-sections as a function of $$p_{\mathrm{T}}$$ are presented for the $${\eta _{{c}}} (1S)$$ and $$\chi _c$$ states in the LHCb acceptance and for $$p_{\mathrm{T}} > 4 {\,\mathrm{GeV}} $$. Next-to-leading-order calculations of the $$p_{\mathrm{T}}$$ dependence of the $$\eta _{{c}} $$ and $$\chi _c$$ production rates in $${b} $$-hadron decays will help to relate the results to conclusions on production mechanisms.

The production rate of the $${\eta _{{c}}} (2S)$$ state in $${b} $$-hadron decays is determined to be

$$\begin{aligned}&{\mathcal {B}} ( b \rightarrow {\eta _{{c}}} (2S) X ) \times {\mathcal {B}} ( {\eta _{{c}}} (2S) \rightarrow \phi \phi )\\&\quad = ( 6.34 \pm 1.81 \pm 0.57 \pm 1.89_{{\mathcal {B}}} ) \times 10^{-7}. \end{aligned}$$

This is the first measurement for inclusive $${\eta _{{c}}} (2S)$$ production rate in $${b} $$-hadron decays and the first evidence for the decay $${\eta _{{c}}} (2S) \rightarrow \phi \phi $$. The production rate as a function of the assumed natural width is given in Fig. 5. These are the first $$\chi _c$$ and $${\eta _{{c}}} (2S)$$ inclusive production measurements, using charmonium decays to a hadronic final state, in the high-multiplicity environment of a hadron machine. In addition, upper limits at $$95 \%$$ ($$90 \%$$) CL on the production rates of the *X*(3872), *X*(3915), and $${\upchi _{{{c}} 2}} (2P)$$ states in $${b} $$-hadron decays are obtained,

$$\begin{aligned}&R^{X(3872)}_{{\upchi _{{{c}} 1}}}< 0.39 \ (0.34),\\  &R^{X(3915)}_{{\upchi _{{{c}} 0}}}< 0.14 \ (0.12)\quad \mathrm{and}\\ &R^{{\upchi _{{{c}} 2}} (2P)}_{{\upchi _{{{c}} 2}}} < 0.20 \ (0.16), \end{aligned}$$

or

$$\begin{aligned} {\mathcal {B}} ( {{b}} \rightarrow X(3872) X ) \times {\mathcal {B}} ( X(3872) \rightarrow \phi \phi )< & {} 4.5 (3.9) \times 10^{-7}, \\ {\mathcal {B}} ( {{b}} \rightarrow X(3915) X ) \times {\mathcal {B}} ( X(3915) \rightarrow \phi \phi )< & {} 3.1 (2.7) \times 10^{-7}, \\ {\mathcal {B}} ( {{b}} \rightarrow {\upchi _{{{c}} 2}} (2P) X ) \times {\mathcal {B}} ( {\upchi _{{{c}} 2}} (2P) \rightarrow \phi \phi )< & {} 2.8 (2.3) \times 10^{-7}. \end{aligned}$$

Masses and natural widths of the $\eta _{{c}}$ and $\chi _c$ states agree with the world averages. The precision of the ${\eta _{{c}}} (1S)$ mass is comparable to the precision of the world average value. The measured ${\eta _{{c}}} (1S)$ mass is in agreement with the LHCb measurement using decays to the ${{p}} {\overline{{{p}}}}$ final states [15].

First evidence for the transition ${{{B}} ^0_{{s}}} \rightarrow \phi \phi \phi$ is reported with a significance of 4.9 standard deviations. Its branching fraction is measured to be

$$\begin{aligned} {\mathcal {B}} ( {{{B}} ^0_{{s}}} \rightarrow \phi \phi \phi ) = ( 2.15 \pm 0.54 \pm 0.28 \pm 0.21_{{\mathcal {B}}} ) \times 10^{-6}. \end{aligned}$$

No resonant structure is observed in the $\phi \phi$ invariant mass distribution. In the ${{{B}} ^0_{{s}}} \rightarrow \phi \phi \phi$ decay, transverse polarization is preferred for the $\phi$ mesons, with an estimate of $f_{\text {T}} > 0.28$ at $95 \%$ CL and the most probable value of $f_{\text {T}} = 0.86$ for the fraction of transverse polarization.

As a by-product of the analysis, the branching fraction $${\mathcal {B}} ( {{{B}} ^0_{{s}}} \rightarrow \phi \phi )$$ is determined to be $${\mathcal {B}} ( {{{B}} ^0_{{s}}} \rightarrow \phi \phi ) = ( 2.18 \pm 0.17 \pm 0.11 \pm 0.14_{f_s} \pm 0.65_{{\mathcal {B}}} ) \times 10^{-5}$$ with a different technique with respect to the previous results [21, 22, 63, 64]. This technique is based on relation of $${{B}} ^0_{{s}} $$ production in $${p} $$ $${p} $$ collisions and $${\eta _{{c}}} (1S)$$ inclusive production rate in $${b} $$-hadron decays, and reconstruction of $${{B}} ^0_{{s}} $$ and $${\eta _{{c}}} (1S)$$ via decays to $$\phi \phi $$. The measurement is consistent with the recent LHCb result [22] and the current world average [14], as well as with theoretical calculations [29, 30, 65].

Finally, using the measurements presented and external input, the ratio of the branching fractions for the $${\eta _{{c}}} (1S)$$ decays to $$\phi \phi $$ and to $${{p}} {\overline{{{p}}}} $$ is determined. The measured $${{B}} ^0_{{s}} $$ and $${\eta _{{c}}} (1S)$$ yields and efficiency ratio, the branching fraction $${\mathcal {B}} ( {{{B}} ^0_{{s}}} \rightarrow \phi \phi ) = (1.84 \pm 0.05 \pm 0.07 \pm 0.11_{f_s / f_d} \pm 0.12_{\mathrm{norm}}) \times 10^{-5}$$ [22], the $${{J}/\uppsi }$$ production rate in $${b} $$-hadron decays $${\mathcal {B}} ( {{b}} \rightarrow {{{J}/\uppsi }} X ) = (1.16 \pm 0.10 ) \%$$ [14], the relative production rates of $${\eta _{{c}}} (1S)$$ and $${{J}/\uppsi }$$ in $${b} $$-hadron decays $$\frac{{\mathcal {B}} ( {{b}} \rightarrow {\eta _{{c}}} (1S) X) \times {\mathcal {B}} ( {\eta _{{c}}} (1S) \rightarrow {{p}} {\overline{{{p}}}})}{{\mathcal {B}} ( {{b}} \rightarrow {{{J}/\uppsi }} X) \times {\mathcal {B}} ( {{{J}/\uppsi }} \rightarrow {{p}} {\overline{{{p}}}})} = 0.302 \pm 0.042$$ [15], the branching fraction $$ {\mathcal {B}} ( {{{J}/\uppsi }} \rightarrow {{p}} {\overline{{{p}}}}) = ( 2.120 \pm 0.029 ) \times 10^{-3}$$ [14], the ratio of fragmentation fractions $$f_s / f_d = 0.259 \pm 0.015$$ [66], and the $${\Lambda } ^0_{{b}} $$ fragmentation fraction $$f_{{{\Lambda } ^0_{{b}}}}$$ momentum dependence from Ref. [67] are used. The ratio of the branching fractions for the $${\eta _{{c}}} (1S)$$ decays to $$\phi \phi $$ and to $${{p}} {\overline{{{p}}}} $$ is determined as

$$\begin{aligned} \frac{{\mathcal {B}} ( {\eta _{{c}}} (1S) \rightarrow \phi \phi )}{{\mathcal {B}} ( {\eta _{{c}}} (1S) \rightarrow {{p}} {\overline{{{p}}}})}= & {} 1.79 \pm 0.14 \pm 0.09 \pm 0.10_{f_s / f_d}\\&\pm 0.03_{f_{{{\Lambda } ^0_{{b}}}}} \pm 0.29_{{\mathcal {B}}}, \end{aligned}$$

where the third uncertainty is related to $f_s / f_d$, the fourth uncertainty is related to $f_{{{\Lambda } ^0_{{b}}}}$, and the fifth uncertainty is related to uncertainties of the production rates and decay branching fractions involved. This value is larger than the value computed from the world average branching fractions given in Ref. [14].

## Footnotes

1 The inclusion of charge-conjugate states is implied throughout.

2 Natural units are used throughout the paper.
